# CircLRBA Promotes Epithelial‐Mesenchymal Transition, Immune Evasion, Chemoimmunotherapy Resistance and Metastasis Through Stabilizing Twist1

**DOI:** 10.1002/advs.202508918

**Published:** 2025-10-13

**Authors:** Xiaosong Wang, Xin Yang, Lei Xing, Hang Chen, Fuming Xie, Bin Wang, Bowen Shi, Yan Yang, Junxia Chen

**Affiliations:** ^1^ Department of Cell Biology and Genetics Chongqing Medical University Chongqing 400016 China; ^2^ Department of breast and thyroid surgery The First Affiliated Hospital of Chongqing Medical University Chongqing 400016 China; ^3^ Institute of Hepatopancreatobiliary Surgery, Chongqing General Hospital Chongqing University Chongqing 401147 China; ^4^ Department of Oncology The Seventh People's Hospital of Chongqing Chongqing 400054 China

**Keywords:** breast cancer, chemoimmunotherapy resistance, circLRBA, epithelial‐mesenchymal transition, immune evasion

## Abstract

Breast cancer (BC) is a malignant tumor with the highest incidence in women. Metastasis is the leading cause of BC‐related death. Circular RNAs (circRNAs) play important roles in cancer progression and metastasis, therefore exploring its specific mechanism in BC metastasis has high value. However, the biological roles and potential mechanism of circRNAs in BC remain unclear. Here, a highly expressed circRNA circLRBA in BC tissues is identified using high‐throughput sequencing, which is associated with pathological stage and poor overall survival. Functional assays show that circLRBA facilitates BC cell proliferation, invasion, migration, docetaxel (DTX) resistance, and inhibits the infiltration of CD8+ T cell in vitro and in vivo. Whereas circLRBA knockdown reveals opposite roles. Mechanistically, transcription factor Zeb1 promotes the generation of circLRBA. Importantly, circLRBA could competitively combine with E3 ubiquitin ligase SPOP to suppress the Twist1 ubiquitination degradation and enhances PD‐L1 transcriptional activity, thus promoting EMT, immune evasion, chemoresistance and BC progression. This study highlights the oncogenic role of circLRBA in BC progression through its binding with SPOP to increases Twist1 stability, suggesting that circLRBA might serve as a promising biomarker and potential therapeutic target for BC.

## Graphical abstract

CircLRBA is highly expressed in breast cancer (BC) and associated with poor overall survival. Zeb1 mediated circLRBA facilitates BC cell proliferation, invasion, docetaxel (DTX) resistance and inhibits the infiltration of CD8+ T cell. CircLRBA could combine with SPOP to suppress the Twist1 ubiquitination degradation and enhances PD‐L1 transcription, thus promoting EMT, immune evasion, chemoresistance and BC progression. This study highlights circLRBA might serve as a promising biomarker and potential therapeutic target for BC.

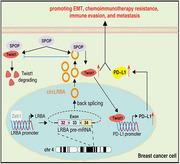

## 1 Introduction

Breast cancer (BC) has surpassed lung cancer to become the tumor with the highest incidence rate among women in 157 countries globally, and is also a leading cause of cancer death in women.^[^ ^1^ ^]^ In the future, the number of newly diagnosed BC patients is expected to increase by ≈3 million cases every year compared with 2020. If the current trends remain unchanged, by 2040, the number of people who die from BC each year will be over 50% higher than it is now, exceeding one million.^[^ ^2^ ^]^ Although clinical methods such as surgery and chemotherapy, endocrine therapy and targeted therapy are broadly used in BC patients, late diagnosis and metastasis are the main causes of death. According to statistics, ≈25% of patients with metastatic cancer have a 5‐year survival rate.^[^ ^3^ ^]^ Therefore, it is urgent to explore the potential mechanisms and effective biomarkers for the metastasis of BC.

CircRNAs, a class of RNA molecules with a single‐stranded covalent closed‐loop structure, play important roles in the occurrence and progression of diseases through various mechanisms, especially in cancer development.^[^ ^4^ ^]^ Due to their tissue‐ and development‐specific expression, evolutionary conservation and high stability, circRNAs may serve as a potential biomarkers for cancer diagnosis, treatment and prognosis.^[^ ^5^ ^]^ Accumulating studies have indicated that circRNAs serve as sponges of miRNAs to modulate gene expression in cancer development and progression through circRNA‐miRNA‐mRNA axis. For example, hsa_circRNA_104348 facilitates hepatocellular carcinoma development via sponging miR‐187‐3p and activating the Wnt/β‐catenin pathway.^[^ ^6^ ^]^ Liu et al. demonstrated that the circular RNA hsa_circ_001783 interacts with miR‐200c‐3p to promote the progression of breast cancer.^[^ ^7^ ^]^ Furthermore, some circRNAs can also act as protein sponges to form circRNA‐protein complexes that regulate the interactions between proteins and downstream target mRNAs or proteins. For instance, circNEIL3 interacts with Nedd4L to degrade YBX1 and inhibits colorectal carcinoma metastasis.^[^ ^8^ ^]^ CircACTN4 regulates the expression of c‐myc through competitively sponging FUBP1, thus promoting the occurrence and development of breast cancer.^[^ ^9^ ^]^ Additionally, a few circRNAs have been reported to function as translation templates of peptides or proteins. Circ‐EIF6 encodes EIF6‐224aa to promote TNBC development through MYH9 stability and Wnt/beta‐catenin pathway activation.^[^ ^10^ ^]^ Moreover, circCAPG encodes CAPG‐171aa, which increases the proliferation and metastasis of TNBC cells via activating the MEKK2‐MEK1/2‐ERK1/2 pathway.^[^ ^11^ ^]^ However, the possibility that circRNAs play important roles in the epithelial‐mesenchymal transition (EMT) and metastasis of BC through other regulatory mechanisms remains largely unknown.

EMT, an evolutionarily conserved developmental program, has been shown to be related to metastasis via increasing the mobility and invasion of cancer cells,^[^ ^12^ ^]^ and over 90% of cancer‐related deaths are caused by metastasis.^[^ ^13^ ^]^ Multiple signaling pathways, such as the TGF signaling pathway, the Notch signaling pathway, and the Wnt/β‐catenin pathway, as well as several transcription factors, including Snail, Zeb, and Twist, have been proved to be involved in the modulation of EMT.^[^ ^14^, ^15^ ^]^ Abnormally expressed Twist (also known as Twist1) causes the loss of intercellular adhesion mediated by E‐cadherin, leading to cell metastasis.^[^ ^16^, ^17^ ^]^ Studies have reported that Twist1 mediates EMT and metastasis in stomach adenocarcinoma,^[^ ^18^ ^]^ gliomas,^[^ ^19^ ^]^ and lung cancer.^[^ ^20^ ^]^ In addition, Guan et al. reported that the CDK1‐mediated phosphorylation of USP29 promoted the stability of Twist1 and EMT in breast cancer.^[^ ^21^ ^]^ The E3 ubiquitin ligase FBXO3 can interact with USP4 to stabilize Twist1, thus facilitating breast cancer metastasis.^[^ ^22^ ^]^ Notably, Twist1‐induced EMT has been implicated in promoting chemoresistance in BC. For example, MUC1‐C binds to Twist1 in TNBC, thus promoting EMT, stemness and paclitaxel resistance.^[^ ^23^ ^]^ Xu et al. reported that DYRK2 enhances docetaxel (DTX) chemoresistance by activating Twist1‐induced EMT.^[^ ^24^ ^]^

PD‐1 is an inducible protein that is expressed on activated T and B cells. Before activation, T cells indicate weak PD‐1 expression, which gradually enhances after antigenic stimulation. PD‐L1, the functional ligand of PD‐1, may act as a molecular “barrier” to protect PD‐L1+ tumor cells from CD8+ T cell‐mediated antitumor immunity. Moreover, a previous study has demonstrated that ectopic expression of Twist1 promotes PD‐L1 transcription, thereby facilitating CD8+ T cell exhaustion and immune evasion.^[^ ^25^ ^]^ Collectively, these findings highlight the pivotal role of Twist1 in cancer progression. However, to date, research on how circRNAs regulate the level of Twist1 to promote EMT and chemoimmunotherapy resistance in BC is lacking.

In the present study, circLRBA was identified through RNA sequencing and found to be highly expressed in BC tissues. The expression of circLRBA was related to pathological stage and survival. Moreover, we demonstrated that Zeb1 facilitates the transcription of the parent gene of circLRBA and circLRBA production. We showed that circLRBA markedly promotes the proliferation, invasion, migration, EMT, metastasis, resistance to DTX and anti‐PD‐L1 immunotherapy of BC cells. Mechanistically, circLRBA could bind to the E3 ubiquitin ligase SPOP to interrupt the binding between SPOP and Twist1 and suppress the degradation of Twist1, and the circLRBA/Twist1 axis could enhance the transcriptional activity of PD‐L1. Taken together, the findings of this study reveal a novel mechanism by which circLRBA promotes EMT, immune evasion, chemoimmunotherapy resistance and metastasis in BC, suggesting that circLRBA might serve as a potential target for therapy and prognosis of BC.

## 2 Results

### 2.1 CircLRBA is Identified and Characterized in BC

**Figure 1:**
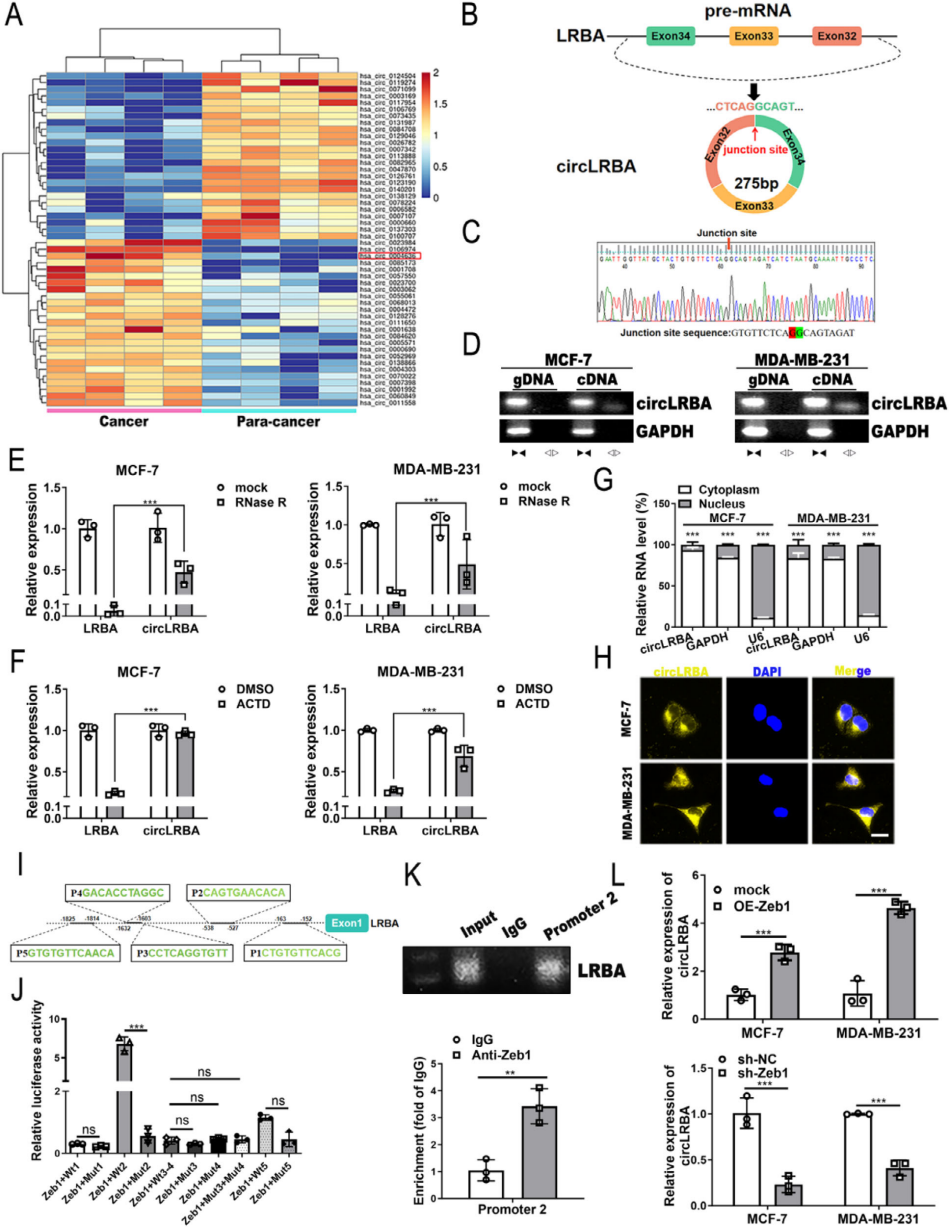
CircLRBA is identified and verified in BC cells. A) Heatmap of the 25 most increased and decreased circRNAs in four pairs of BC and paracancerous tissues. B) Schematic diagram showing that circLRBA (hsa_circ_0004636) is produced by back‐splicing of exon 32 to exon 34 of the LRBA gene. C) Sanger sequencing verified the junction site of back‐splicing of circLRBA. D) The gDNA and cDNA of circLRBA and GAPDH in MCF‐7 cells were amplified by PCR with convergent and divergent primers, respectively. E) The relative expression of circLRBA and LRBA in MCF‐7 and MDA‐MB‐231 cells treated with or without RNase R was determined by qRT‒PCR. F) The relative expression of circLRBA and LRBA in BC cells treated with DMSO or actinomycin D was analyzed using qRT‐PCR. G) The relative expression of circLRBA in the cytoplasm and nucleus was detected by Cytoplasmic/Nuclear fractionation. U6 and GAPDH were used as the nuclear and cytoplasmic controls, respectively. H) The subcellular localization of circLRBA in BC cells was observed using FISH (magnification, ×400; scale bar, 50 µm). I) Schematic model showing the hypothetical sites of Zeb1 in the LRBA gene promoter region. J) A luciferase reporter assay was used to detect the binding between putative sites in the LRBA gene promoter region and Zeb1 in MCF‐7 cells cotransfected with the indicated luciferase reporter vectors and overexpressing Zeb1 plasmids. K) ChIP‒PCR analysis showed that Zeb1 was enriched in the LRBA gene promoter region in MCF‐7 cells, with anti‐Zeb1 and IgG used as controls. L) qRT‒PCR was conducted to analyze the expression level of circLRBA in BC cells after upregulation or downregulation of Zeb1. The data are presented as the mean ± SDs of at least three independent experiments. *p* < 0.05 was considered statistically significant. ns, not significant. **p < 0.01, ***p < 0.001. One‐way ANOVA (E, F and J) and Student's *t*‐test (G, K and L) were used to determine statistical significance.

To explore the roles of circRNAs in the progression of BC, we carried out RNA sequencing of total RNA from 4 pairs of BC and adjacent normal breast tissues. |Fold change|≥2 and *p* < 0.05 were used as cutoff criteria to screen differential expression circRNAs, and 77 differentially expressed circular RNAs were identified, 39 of which were upregulated and 38 of which were downregulated in BC tissues. **Figure** **1**A shows the top 25 most upregulated and top 25 most downregulated circRNAs, among which hsa_circ_0004636 was prominently elevated in BC tissues. We named hsa_circ_0004636 circLRBA because it derives exons 32 to 34 of the LRBA gene (Figure 1B). The back‐splicing site of circLRBA was identified via Sanger sequencing (Figure 1C). Moreover, to verify the covalently closed circular structure of circLRBA, we performed RT‒PCR with convergent and divergent primers. The results revealed amplification of circLRBA by divergent primers (but not convergent primers) in cDNA from MCF‐7 and MDA‐MB‐231 cells exclusively (Figure 1D). GAPDH was used as a negative control. CircLRBA was more resistant to RNase R digestion and Actinomycin D (ACTD) treatment than was LRBA mRNA in BC cells (Figure 1E,F). Furthermore, the results of the cytoplasmic/nuclear fractionation assay revealed that circLRBA was enriched mainly in the cytoplasm of BC cells (Figure 1G). FISH also revealed that circLRBA was localized mainly in the cytoplasm of BC cells (Figure 1H). These data suggested that circLRBA has a circular structure.

Using the JASPAR (http://jaspar.genereg.net) database, we predicted that the transcription factor Zeb1 associated with EMT might bind to the promoter of the LRBA gene and regulate the expression of the LRBA gene. As shown in Figure 1I, five putative sites in the LRBA gene promoter region (P1 to P5) were used to verify the binding between Zeb1 and the LRBA gene promoter. The results of dual‐luciferase reporter assays revealed that Zeb1 markedly increased the luciferase activity of the wild‐type promoter 2 (WT2) but not the mutant promoter 2 (Mut2) (Figure 1J). RT‒PCR and ChIP‐qPCR assays further demonstrated the binding of Zeb1 to the promoter 2 site of the LRBA gene promoter in BC cells (Figure 1K; Figure S1, Supporting Information). To explore the effect of Zeb1 on circLRBA expression in BC cells, we synthesized Zeb1 overexpression and knockdown plasmids and tested their efficiency (Figure S2A,B, Supporting Information) in BC cells. The results of qRT‒PCR indicated that the overexpression of Zeb1 significantly increased circLRBA expression, whereas the depletion of Zeb1 reduced circLRBA levels (Figure 1L), which suggested that Zeb1 could facilitate the expression of circLRBA at the transcriptional level.

### 2.2 CircLRBA is Overexpressed in BC and is Associated with Pathological Staging and Prognosis

**Figure 2:**
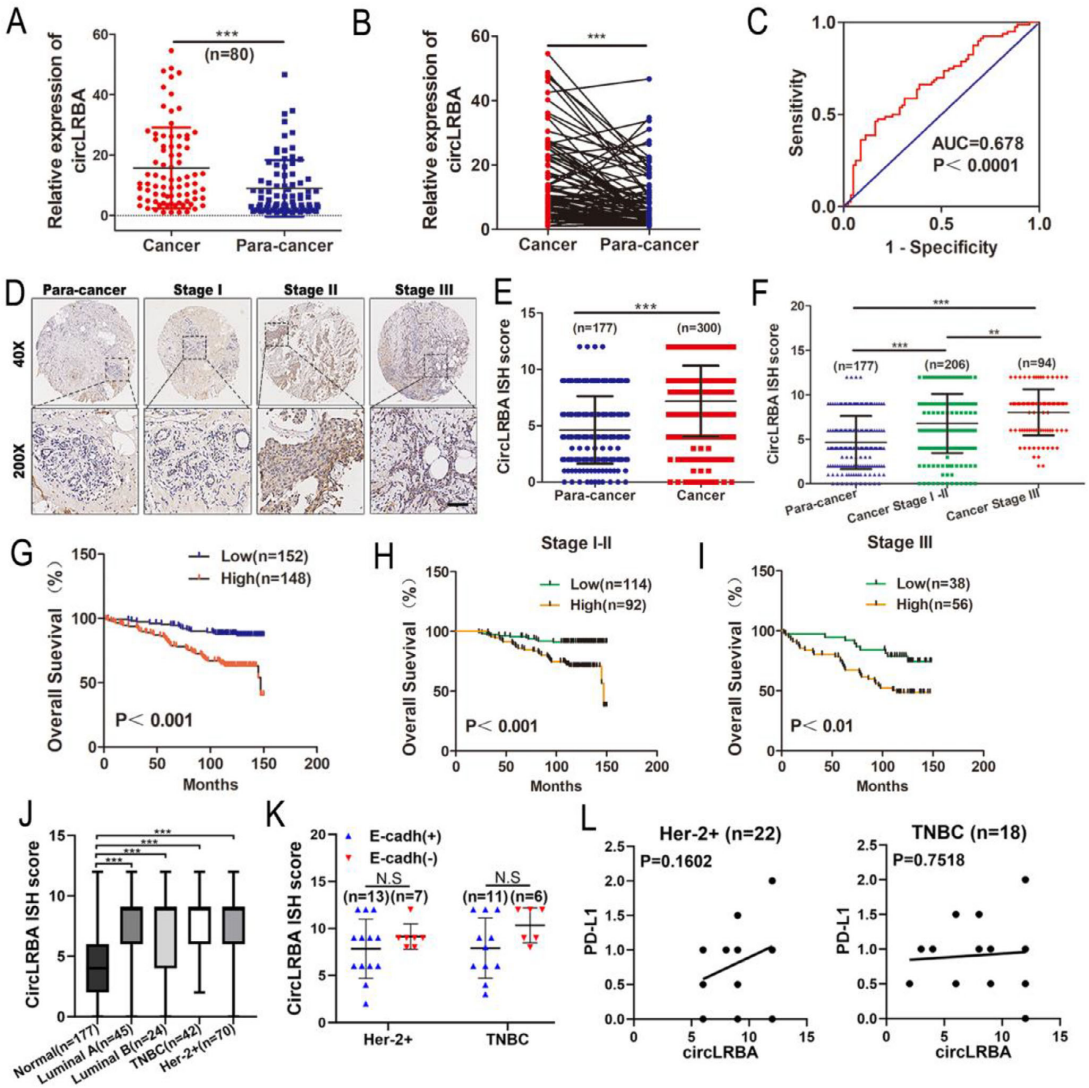
CircLRBA is upregulated and associated with pathological stage and poor prognosis in BC patients. A,B) The relative expression level of circLRBA was determined in 80 pairs of BC tissues and paracancerous tissues by qRT‐PCR. C) ROC curve used to evaluate the diagnostic value of circLRBA for BC. D) Representative images showing the expression of circLRBA with ISH staining of the TMA (scale bar, 100 µm). E) ISH scores for circLRBA are shown for BC and paracancerous tissues on the TMA. F) ISH scores for circLRBA are shown for paracancerous tissues and BC tissues at different stages on the TMA. G) Kaplan–Meier survival curves showing that patients in the high‐expression group had poorer prognoses than those in the low‐expression group did. BC patients were divided into two groups on the basis of the median expression of circLRBA according to the ISH score. H) Kaplan–Meier survival curves showing the survival prognosis of patients in the circLRBA low‐ and high‐expression groups with stage I and II disease. I) Kaplan–Meier survival curves showing the survival prognosis of patients in the circLRBA low‐ and high‐expression groups among stage III patients. J) The expression of circLRBA in different subtypes of breast cancer. K) The relationship between circLRBA expression and E‐cadherin levels was analyzed in HER2‐positive BC and TNBC. L) The association of circLRBA expression with PD‐L1 levels was evaluated via Pearson correlation in both HER2‐positive BC and TNBC. The data are presented as the mean ± SDs of at least three independent experiments. *p* < 0.05 was considered statistically significant. N.S, not significant. **p < 0.01, ***p < 0.001. Student's *t*‐test (A, B, E and K), One‐way ANOVA (F and J), log‐rank tests (G, H and I) and Pearson correlation analysis (L) were used to determine statistical significance.

To assess the clinical significance of circLRBA in BC, we detected the expression level of circLRBA in 80 pairs of BC and paracancerous tissues via qRT‒PCR. The data indicated that circLRBA was highly expressed in BC tissues (**Figure** **2**A,B). The diagnostic performance of circLRBA for BC was evaluated via receiver operating characteristic (ROC) curves. The area under the curve (AUC) of circLRBA was 0.678, and when the cutoff value was 3.66, the sensitivity and specificity were 0.838 and 0.462, respectively (Figure 2C). Correlations between circLRBA expression and the clinical characteristics of 80 BC patients were analyzed, and the results indicated that circLRBA expression was associated with T stage and TNM stage (**Table** **1**). Subsequently, tissue microarrays (TMAs) containing 300 BC tissues and 177 normal breast tissues were stained to detect the expression of circLRBA by ISH using a specific biotin‐labeled probe of circLRBA. The results revealed that circLRBA was significantly overexpressed in BC tissues compared with normal breast tissues (Figure 2D,E). Furthermore, circLRBA expression was positively correlated with the T stage, N stage and TNM stage of BC patients (**Table** **2**; Figure 2F). Survival curves were drawn via the Kaplan–Meier method to estimate the prognosis of BC patients. The data showed that patients with high expression of circLRBA had shorter overall survival than did those with low expression of circLRBA. Additionally, TNM stage I‐II and stage III patients with high levels of circLRBA had worse prognoses than low level circLRBA patients did (Figure 2G–I). Cox proportional hazard analysis was used to evaluate the prognostic value of circLRBA for BC. The results showed that circLRBA could be an independent predictor of poor prognosis in BC patients (**Table** **3**). Next, we examined the associations between circLRBA expression and different molecular subtypes of breast cancer. As shown in Figure 2J, circLRBA was highly expressed across all subtypes. However, in the Her‐2‐positive and TNBC subtypes, its expression was not significantly correlated with either E‐cadherin or PD‐L1 levels (Figure 2K,L). In brief, the above findings demonstrate that circLRBA is highly expressed in BC and might be a novel biomarker for BC prognosis.

**Table 1:** Correlation between circLRBA expression and clinicopathological features in 80 BC patients (cohort 1).

Characteristic		All cases	circLRBA		Chi‐square	*p* value
			low high			
All cases		80	40	40		
Age	˂50	39	20	19	0.050	0.823
	≥50	41	20	21		
Grade	I‐II	63	31	32	0.075	0.785
	III	17	9	8		
T stage	T1	42	27	15	7.218	0.007**
	T2‐3	38	13	25		
N stage	N0‐1	64	31	33	0.313	0.576
	N2‐3	16	9	7		
TNM stage	I‐II	61	36	25	8.352	0.004**
	III	19	4	15		
Menopausal	Premenopausal	35	18	17	2.100	0.147
	Postmenopausal	45	22	23		

**p ˂ 0.01

**Table 2:** Correlation between circLRBA expression and clinicopathological features in 300 BC patients (cohort 2).

Characteristic		All cases	circLRBA		Chi‐square	*P* value
			low high			
All cases	300	152		148		
Age	˂52	135	69	66	0.019	0.889
	≥52	165	83	82		
Grade	I‐II	252	132	120	1.852	0.174
	III	48	20	28		
T stage	T1	125	75	50	7.468	0.006**
	T2‐3	175	77	98		
N stage	N0‐1	210	116	94	5.852	0.016*
	N2‐3	90	36	54		
TNM stage	I‐II	206	114	92	5.744	0.017*
	III	94	38	56		

*p ˂ 0.05, **p < 0.01

**Table 3:** Univariate and multivariate Cox regression analysis of circLRBA and survival in 300 BC patients.

Clinical variables	Univariate analysis		*p* value	Multivariate analysis		*p* value
	HR 95%CI			HR 95%CI		
Age(≥52 vs.˂52)	1.867	1.169–2.982	0.009	1.842	1.149–2.952	0.011*
Grade(I‐II vs III)	2.065	1.224–3.486	0.007	1.590	0.938–2.698	0.085
T stage(T1 vs.T2/3)	1.206	0.754–1.930	0.434			
N stage(N0‐1 vs N2‐3)	2.458	1.562–3.868	0.001	2.353	1.493–3.710	0.001**
TNM stage (I ‐II vs III)	1.711	0.852–3.436	0.131			
circLRBA (low vs. high)	3.553	2.111–5.982	0.001	3.200	1.895–5.403	0.001**

Abbreviations: HR hazard ratio, CI confidence interval, *p ˂ 0.05, **p ˂ 0.01

### 2.3 CircLRBA Facilitates the Proliferation, Invasion, Migration and EMT of BC Cells In Vitro

**Figure 3:**
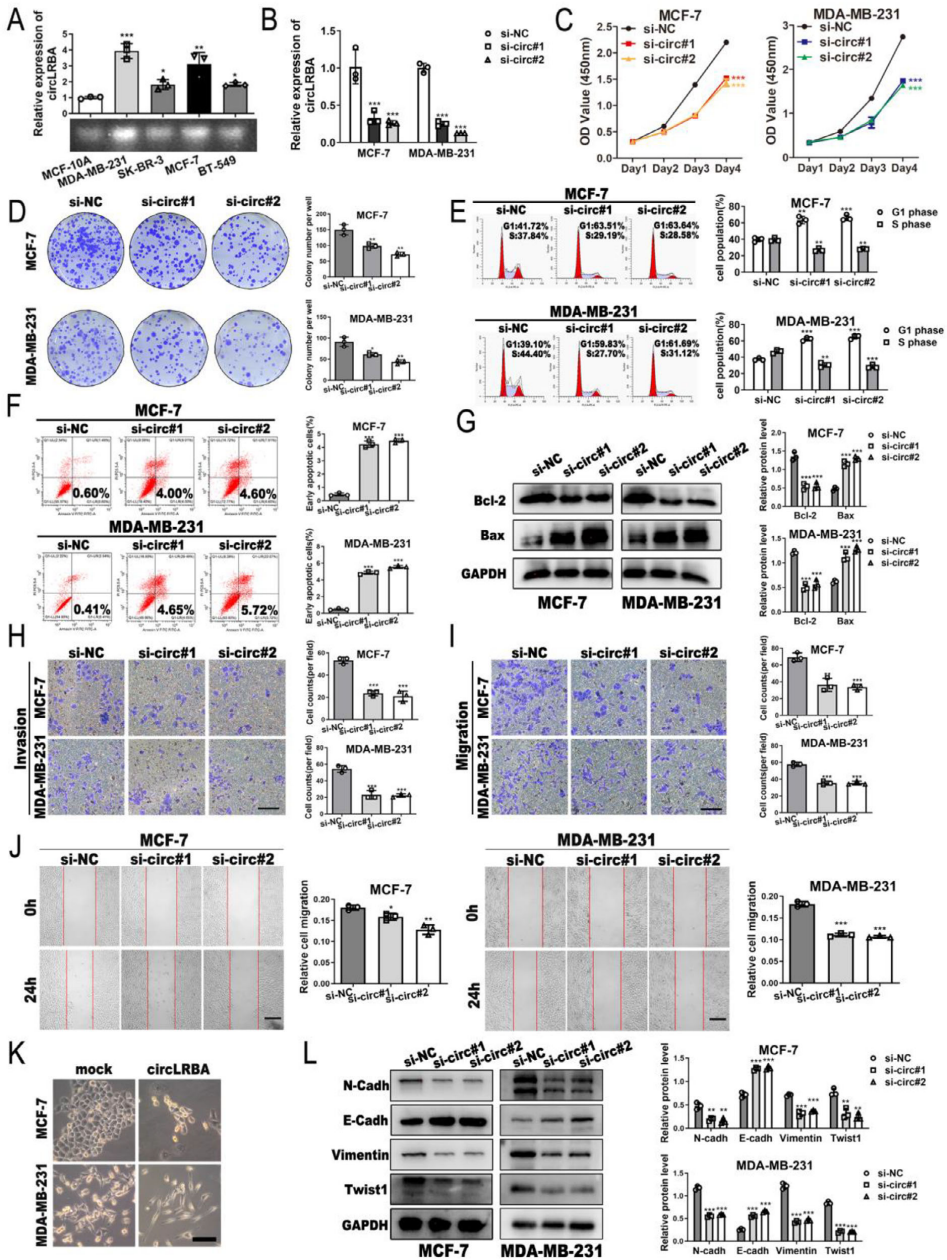
CircLRBA increases the proliferation, invasion and migration of BC cells in vitro. A) The relative expression of circLRBA in various BC cell lines was analyzed by qRT‒PCR and RT‒PCR. B) The knockdown levels of circLRBA were detected after BC cells were transfected with siRNAs. C,D) CCK‐8 (C) and colony formation (D) assays were used to detect the proliferative ability of BC cells after circLRBA was knocked down. E,F) Cell cycle distribution (E) and early apoptosis (F) were examined using flow cytometry after circLRBA depletion. G) Apoptosis‐related protein levels were assessed by western blotting. H–J) The invasion and migration abilities of BC cells after circLRBA downregulation were detected using Transwell assays with (H; invasion assay) or without Matrigel (I; migration assay) and a wound healing (J) assay. (Magnification: Transwell assays, ×100; wound healing assay, ×50; scale bar: 200 µm for all) K) Representative morphology images of BC cells transfected with circLRBA overexpression plasmids (magnification, ×200; scale bar, 100 µm). L) The expression levels of EMT‐related proteins were detected via western blotting in BC cells after circLRBA was knocked down. The data are presented as the mean ± SDs of at least three independent experiments. *p* < 0.05 was considered statistically significant. *p < 0.05, **p < 0.01, ***p < 0.001. One‐way ANOVA (A‐J and L) was used to determine statistical significance.

To investigate the roles of circLRBA in BC progression, we assessed the differential expression of circLRBA between BC cell lines (MCF‐7, SK‐BR‐3, MDA‐MB‐231 and BT‐549) and the normal breast epithelial cell line MCF‐10A. The results indicated that the expression of circLRBA was higher in MCF‐7 and MDA‐MB‐231 cells than in MCF‐10A cells (**Figure** **3**A); thus, these two BC cell lines were utilized in subsequent studies. Next, we constructed circLRBA‐overexpressing plasmids and two circLRBA siRNAs, and the qRT‒PCR results showed that circLRBA was significantly upregulated or downregulated in BC cells transfected with the indicated plasmids or siRNAs (Figure S3A, Supporting Information; Figure 3B). However, overexpression or knockdown of circLRBA did not affect the LRBA mRNA level (Figure S3B, Supporting Information). The results of the CCK‐8 and colony formation assays revealed that the upregulation of circLRBA promoted BC cell proliferation (Figure S3C,D, Supporting Information), whereas the knockdown of circLRBA inhibited BC cell viability (Figure 3C,D). Moreover, flow cytometry analysis indicated that circLRBA depletion triggered G1 arrest in BC cells (Figure 3E). Knockdown of circLRBA led to an increase in the early apoptosis rate of BC cells (Figure 3F), and western blot results showed that circLRBA depletion led to increased protein levels of Bax and decreased expression of Bcl‐2 in BC cells (Figure 3G). We then examined the invasion and migration of BC cells using a transwell assay with and without matrigel and a wound healing assay. The results revealed that circLRBA knockdown significantly suppressed the invasion and migration ability of BC cells, whereas circLRBA overexpression had the opposite effect (Figure 3H–J; Figure S3E–G, Supporting Information). To confirm that delayed wound closure in circular LRBA knockdown MDA‐MB‐231 cells was caused by impaired migration rather than decreased survival, a CCK‐8 assay was performed in serum‐free culture medium. The results showed that the expression of circLRBA did not affect cell survival at 12 and 24 h, suggesting that this delay was due to damage to migration (Figure S4, Supporting Information). Moreover, BC cells in the circLRBA‐overexpressing group displayed a spindle‐shaped morphology, whereas those in the control group were cuboidally shaped epithelial‐like cells (Figure 3K). The western blot results showed that circLRBA knockdown markedly decreased the levels of Twist1 and the EMT‐associated proteins N‐cadherin and vimentin but increased the expression of E‐cadherin (Figure 3L), whereas the upregulation of circLRBA had the opposite effects (Figure S3H, Supporting Information). Collectively, our data suggest that circLRBA might function as an oncogene in BC cells.

### 2.4 CircLRBA Promotes the Growth and Metastasis of BC Cells In Vivo

**Figure 4:**
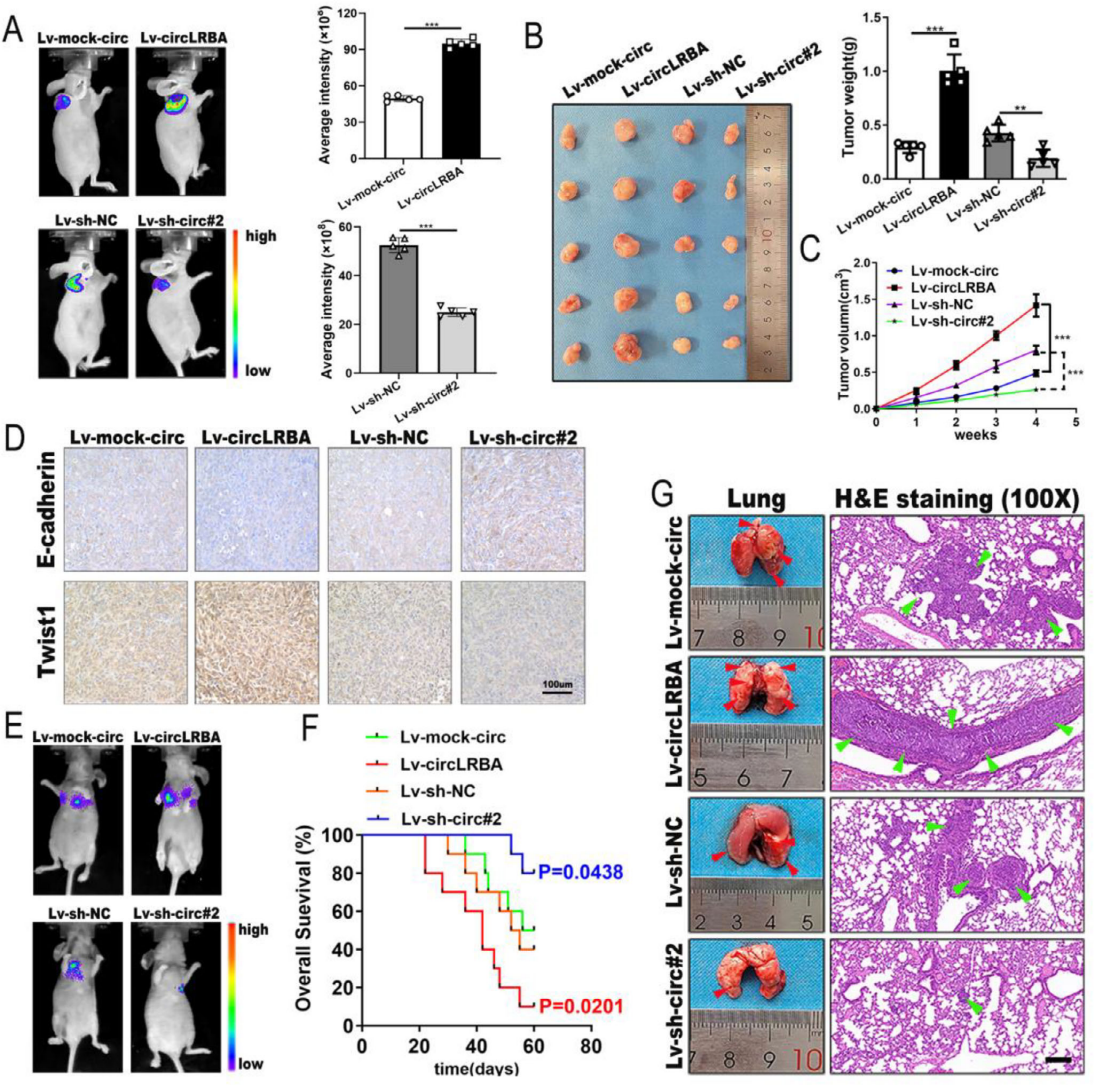
CircLRBA promotes the growth and metastasis of BC cells in vivo. A) Bioluminescent imaging (BLI) was used to detect the growth of MDA‐MB‐231 cells infected with the indicated lentivirus in tumor‐bearing mice (n = 5 per group). B) Images of xenograft tumors in each group and tumor weights are shown (n = 5 per group). C) The tumor volumes were calculated once a week, and the growth curves were plotted (n = 5 per group). D) IHC staining was used to investigate the expression levels of E‐cadherin and Twist1 in the xenograft tumor tissues (magnification, ×200; scale bar, 100 µm). E) Representative bioluminescence imaging (BLI) was used to detect metastasis after injecting MDA‐MB‐231 cells infected with circLRBA‐knockdown or circLRBA‐overexpressing lentivirus into the tail vein of mice for 4 weeks (n = 5 per group). F) Kaplan–Meier survival curves of tumor‐bearing mice injected with BC cells via the tail vein were plotted (n = 10 per group). G) Representative photographs of metastatic nodules on the surface of the lungs and H&E staining of lung metastases are shown (n = 5 per group; magnification, ×100; scale bar, 200 µm). The data are presented as the mean ± SDs of at least three independent experiments. *p* < 0.05 was considered statistically significant. *p < 0.05, **p < 0.01, ***p < 0.001. Student's *t‐*test (A, B and C) and log‐rank tests (F) were used to determine statistical significance.

To evaluate the effects of circLRBA on tumor growth and metastasis in vivo, MDA‐MB‐231 cells infected with the indicated lentiviral vectors were inoculated into 4‐week‐old BALB/c female nude mice to establish a subcutaneous xenograft tumor model and an experimental pulmonary metastasis model by subcutaneous injection or tail vein injection. 4 weeks after subcutaneous injection, circLRBA significantly increased the size of the tumor xenografts according to bioluminescence imaging (BLI), whereas circLRBA silencing inhibited tumor growth (**Figure** **4**A). Compared with those in the control groups, the tumor weights and volumes were greater in the circLRBA‐overexpressing group, whereas the tumor weights were lower in the circLRBA knockdown group (Figure 4B,C). In addition, IHC staining of mice tumor sections revealed that overexpressing circLRBA could increase the expression of Twist1 and reduce the level of E‐cadherin, whereas circLRBA silencing led to low expression of Twist1 and high expression of E‐cadherin (Figure 4D). Moreover, the metastases were observed via bioluminescent imaging (BLI). The results revealed stronger and stronger bioluminescent signals in the circLRBA overexpressing group than in the control group, whereas the mice in the circLRBA knockdown group had fewer bioluminescent metastases than did those in the control group (Figure 4E). To further investigate the effect of circLRBA on the overall survival rate, we recorded the death date of tumor‐bearing mice treated with tail vein injection and calculated the survival rate. The results showed that circLRBA expression was negatively correlated with the overall survival of the mice (Figure 4F). H&E staining indicated that more pulmonary metastatic nodules in mice with circLRBA overexpression than in control mice, whereas the circLRBA downregulation group had less lung metastasis (Figure 4G). In brief, these results further revealed that circLRBA could play an oncogenic role in the development and metastasis of BC.

### 2.5 CircLRBA Directly Binds with the E3 Ubiquitin Ligase SPOP

**Figure 5:**
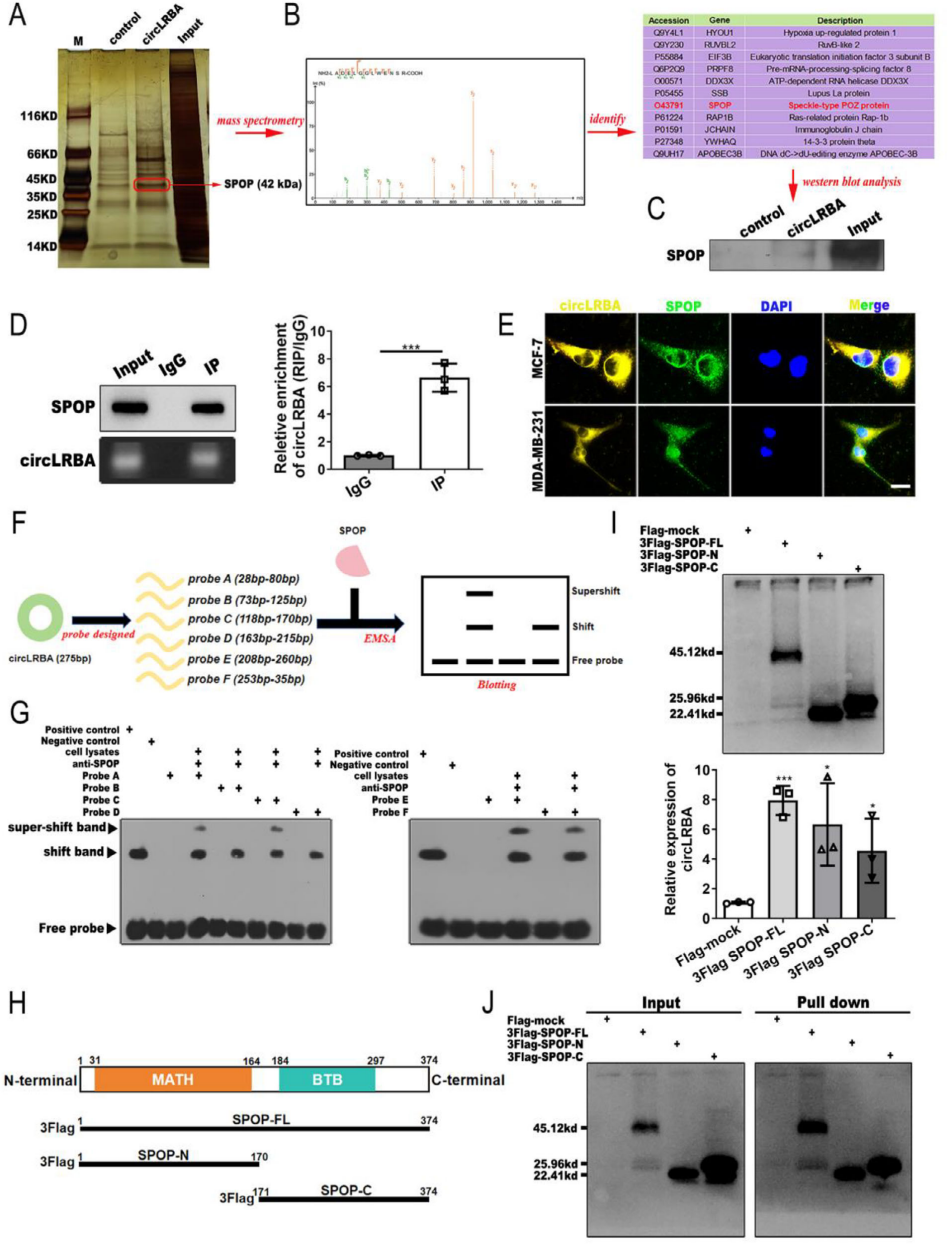
CircLRBA interacts with the E3 ubiquitin ligase SPOP. A) The proteins pulled down by the biotin‐labeled probe specific for circLRBA and the control probe are indicated by silver staining. B) RNA pull‐down proteins were identified via mass spectrometry analysis. C) The interaction between circLRBA and SPOP was confirmed by western blotting. D) The binding between circLRBA and SPOP was verified by RIP using anti‐SPOP and the IgG control. E) The subcellular localization of circLRBA and SPOP in BC cells was determined with FISH and IF (magnification, ×400; scale bar, 50 µm). F) Schematic diagram of specific circLRBA probes used for the EMSA. G) EMSA was performed to detect the exact binding region of circLRBA and SPOP. The supershifted bands indicate SPOP antibody‐bound complexes, confirming specific binding to circLRBA probes. H) Schematic representation of full‐length and truncated segments of the SPOP protein. I) A RIP assay was performed to detect the interaction region of SPOP and circLRBA in MCF‐7 cells transfected with 3×Flag‐tagged full‐length and truncated segments of SPOP plasmids with an anti‐Flag antibody. The precipitated proteins and RNAs were examined by western blotting and qRT‒PCR. J) RNA pull‐down was performed using a specific probe for circLRBA in MCF‐7 cells transfected with full‐length or truncated SPOP, followed by western blotting. The data are presented as the mean ± SDs of at least three independent experiments. *p* < 0.05 was considered statistically significant. *p < 0.05, ***p < 0.001. Student's *t*‐test (D right panel) and One‐way ANOVA (I bottom panel) were used to determine statistical significance.

To elucidate the mechanism of circLRBA in BC development, we performed an RNA pull down assay in MCF‐7 cells to detect binding proteins with circLRBA utilizing a biotin‐labeled circLRBA‐specific probe (**Figure** **5**A). These bound proteins were subsequently identified via mass spectrometry, and the results showed that SPOP was one of the proteins specifically adsorbed by circLRBA (Figure 5B). Previous studies have shown that SPOP can bind to Twist1, leading to the ubiquitination and degradation of Twist1.^[^ ^26^ ^]^ Therefore, we focused on the interaction between circLRBA and SPOP in further studies. The products pulled down by the circLRBA probe were detected by western blot using an anti‐SPOP antibody. The results confirmed the binding of circLRBA and SPOP (Figure 5C). Subsequently, RIP assays were performed via western blotting with antibodies specific for SPOP and primers specific for circLRBA via RT‒PCR and qRT‒PCR to further validate the interaction between SPOP and circLRBA in BC cells (Figure 5D; Figure S5, Supporting Information). A FISH‐IF costaining assay revealed that circLRBA and SPOP were colocalized in the cytoplasm of BC cells, which provided evidence for their interaction (Figure 5E). Notably, circLRBA expression had no effect on the mRNA or protein levels of SPOP (Figure S6A,B, Supporting Information). To explore the precise interaction between circLRBA and SPOP, we designed and synthesized six biotin‐labeled specific probes (A‐F) for EMSA on the basis of the structure of circLRBA, including 28 to 80 bp fragment, 73 to 125 bp fragment, 118 to 170 bp fragment, 163 to 215 bp fragment, 208 to 260 bp fragment, and 253 to 35 bp fragment of circLRBA (Figure 5F). EMSA assay showed that the specific probes A, C, E and F could interact with SPOP (Figure 5G). Furthermore, we constructed Flag‐tagged full‐length SPOP, Flag‐tagged N‐terminal SPOP with a MATH domain (aa 1–170) and Flag‐tagged C‐terminal SPOP with a BTB domain (aa 171–374) vectors to determine the binding region of circLRBA to SPOP (Figure 5H). RIP assays were carried out in MCF‐7 cells transfected with the above plasmids, and the results indicated that both regions of SPOP were bound to circLRBA (Figure 5I). Additionally, pull‐down assays confirmed that circLRBA could interact with both the N‐terminal region and the C‐terminal region of SPOP (Figure 5J). Overall, these results demonstrated that circLRBA could directly bind with SPOP.

### 2.6 SPOP Suppresses EMT and Metastasis by Promoting Twist1 Ubiquitination

**Figure 6:**
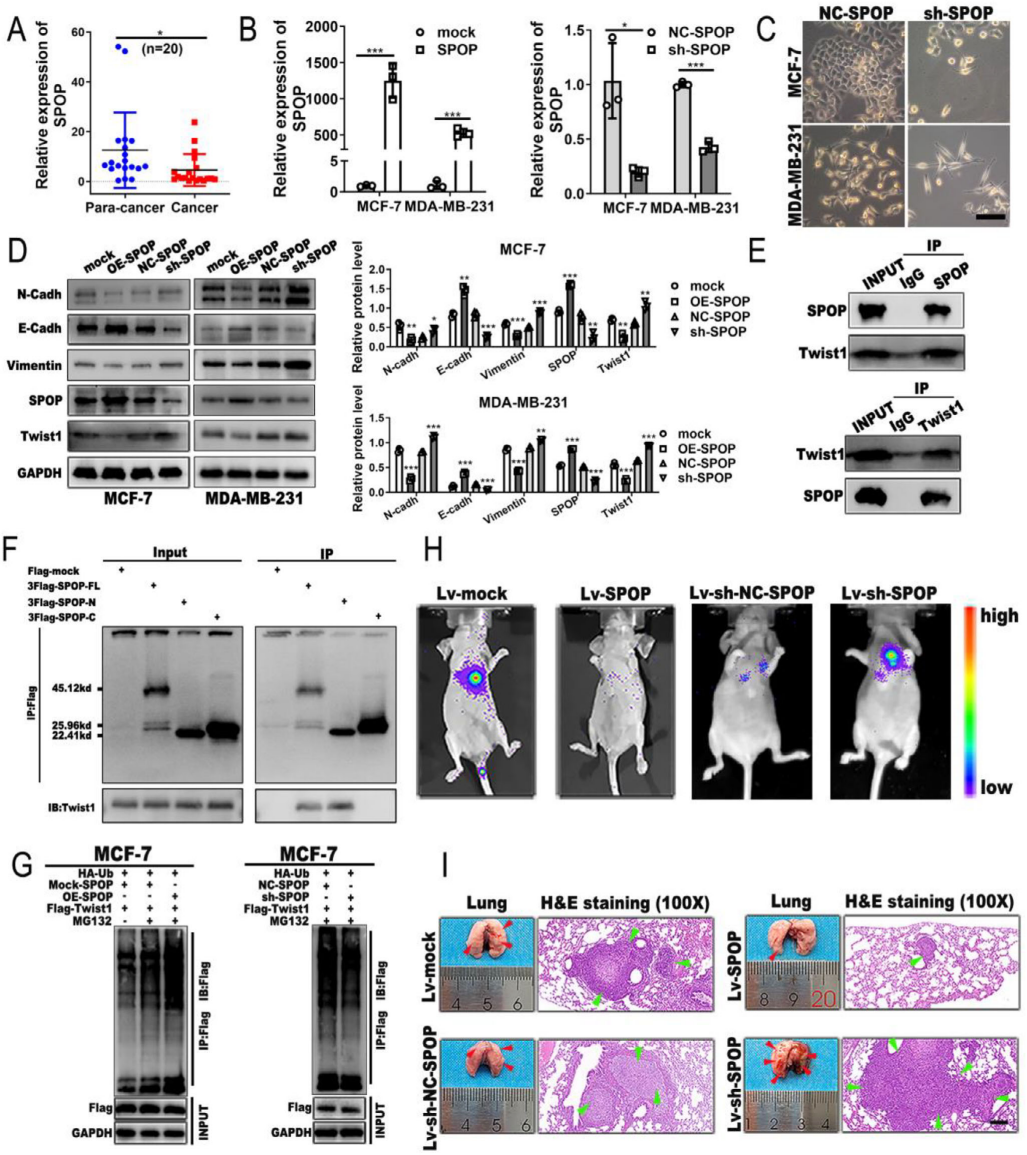
SPOP inhibits the EMT and metastasis of BC by promoting Twist1 ubiquitination. A) The expression of SPOP was determined in BC tissues by qRT‐PCR. B) The expression of SPOP in BC cells transfected with SPOP knockdown or SPOP overexpressing plasmids was determined with qRT‐PCR. C) Representative morphology images of BC cells after SPOP depletion (magnification, ×200; scale bar, 100 µm). D) The expression levels of EMT‐related proteins in BC cells after the upregulation or downregulation of SPOP were detected by western blotting. E) The interaction of SPOP and Twist1 was determined by Co‐IP using anti‐SPOP and anti‐Twist1 antibodies, respectively. F) The binding region of SPOP and Twist1 was detected with Co‐IP via anti‐Flag in MCF‐7 cells transfected with the full‐length N‐terminal domain and C‐terminal domain of SPOP. G) The effect of SPOP expression on the ubiquitination of Twist1 was evaluated using Co‐IP and western blotting in MCF‐7 cells treated with or without 20 µM MG132 for 12 h after transfection for 48 h. H) Representative bioluminescence imaging (BLI) was used to detect metastasis after injecting MDA‐MB‐231 cells infected with the indicated vectors into the tail vein of mice for 4 weeks (n = 5 per group). I) Representative images of metastatic nodules on the surface of the lungs and H&E staining of lung metastases after SPOP overexpression or downregulation are shown (n = 5 per group; magnification, ×100; scale bar, 200 µm). The data are presented as the mean ± SDs of at least three independent experiments. *p* < 0.05 was considered statistically significant. *p < 0.05, **p < 0.01, ***p < 0.001. Student's *t*‐test (A and B) and One‐way ANOVA test (D right panel) were used to determine statistical significance.

To evaluate the role of SPOP in BC, we carried out qRT‒PCR to detect the expression of SPOP, and the results revealed that SPOP was significantly downregulated in BC tissues compared with matched normal tissues (**Figure** **6**A). We subsequently constructed SPOP‐overexpressing plasmids and SPOP shRNA vectors to explore the effects of SPOP on BC (Figure 6B). We found that SPOP depletion caused morphological changes in BC cells from epithelial cells to mesenchymal cells (Figure 6C). Moreover, the western blot results indicated that increased expression of SPOP led to an increase in E‐cadherin protein expression and a decrease in N‐cadherin, vimentin and Twist1 protein levels, whereas downregulation of SPOP had the opposite effect (Figure 6D). To elucidate how SPOP affects the expression of Twist1, co‐IP assays were carried out with anti‐Twist1 or anti‐SPOP antibodies, and the results revealed that SPOP could bind to Twist1 in MCF‐7 cells (Figure 6E). A deletion‐mapping assay with Flag‐tagged full‐length SPOP and its N‐ or C‐terminal truncated mutants using anti‐Flag antibodies showed that the N‐terminal region of SPOP from amino acids 1 to 170 might interact with Twist1, rather than the C‐terminus of SPOP (Figure 6F). We then performed co‐IP and western blot assays to further observe the effect of SPOP on Twist1 ubiquitination. The results revealed that the upregulation of SPOP promoted protein degradation of Twist1, but the knockdown of SPOP diminished the ubiquitination of Twist1 (Figure 6G), suggesting that SPOP decreases the protein level of Twist1 in BC through the promotion of Twist1 ubiquitination. Next, we determined the role of SPOP in BC metastasis. MDA‐MB‐231 cells infected with the indicated vectors were injected into the tail vein of female nude mice. Bioluminescence imaging revealed that the overexpressed SPOP group exhibited weak signals, whereas stronger and more extensive tumor cell metastasis signals were detected in the SPOP‐downregulated group (Figure 6H). Lung tissues from sacrificed mice were sliced and subjected to H&E staining. Compared with those in the control group, more metastatic lung nodules from mice were found in the SPOP depletion group, and fewer nodules were observed in the SPOP‐overexpressing group (Figure 6I). In summary, these findings showed that SPOP could ubiquitinate and destabilize Twist1, thus suppressing the EMT and metastasis of BC cells.

### 2.7 CircLRBA Promotes Invasion, Migration and EMT in BC Cells through the Suppression of Twist1 Degradation Mediated by SPOP

**Figure 7:**
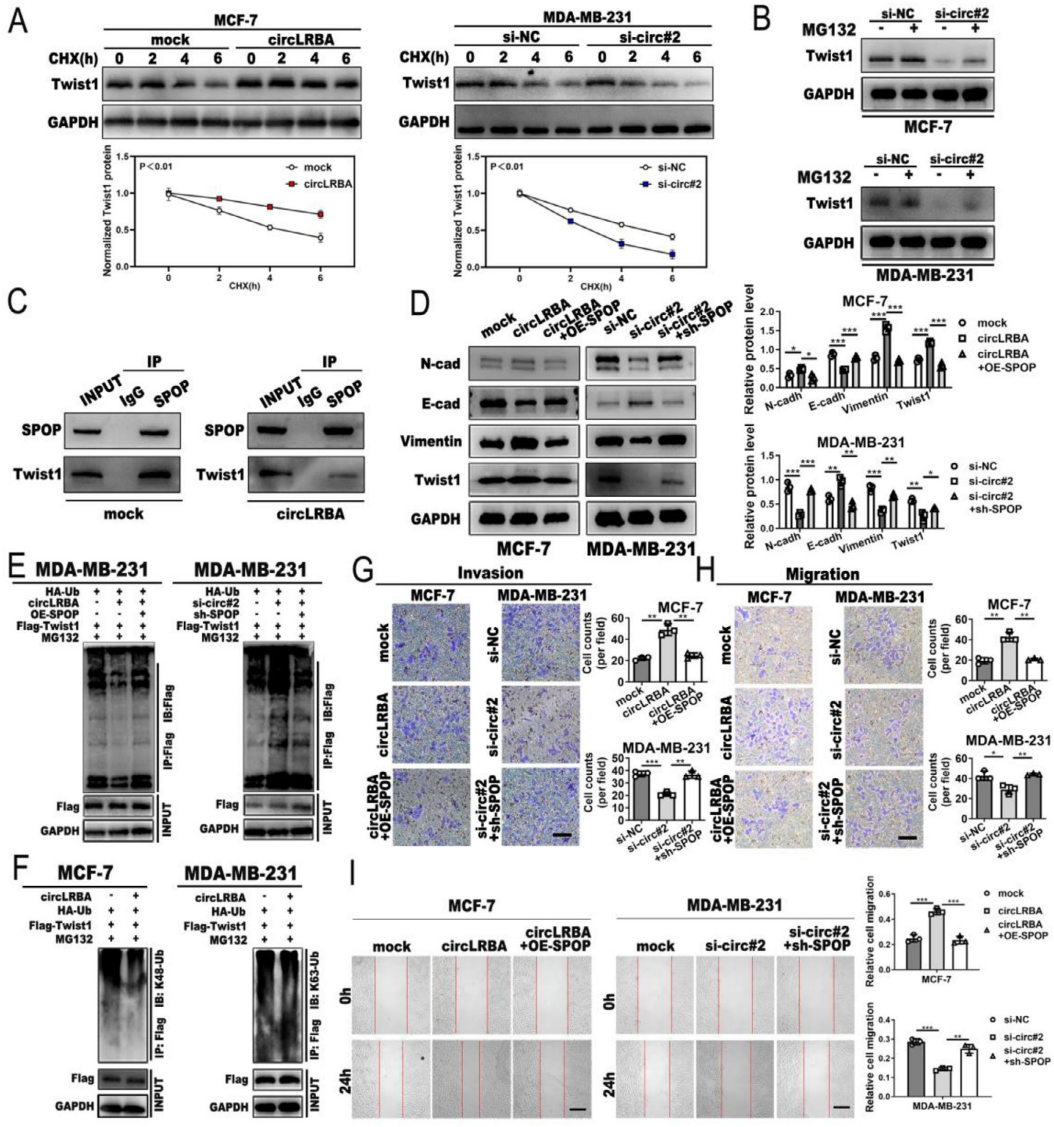
CircLRBA facilitates the invasion, migration and EMT of BC cells by inhibiting SPOP‐mediated Twist1 degradation. A) A CHX chase assay was performed to examine the effect of circLRBA on Twist1 stability. B) Twist1 protein levels were analyzed by western blotting in circLRBA‐knockdown BC cells treated with or without MG132 (20 µM) for 12 h. C) A Co‐IP assay was used to detect the effect of circLRBA on the binding of SPOP to Twist1. D) Western blotting was used to analyze the expression of EMT‐related proteins in BC cells after transfection or cotransfection with the indicated vectors or siRNAs. E) Ubiquitination levels of Twist1 were detected via co‐IP and western blotting in MDA‐MB‐231 cells treated with MG132 after transfection with the indicated plasmids. F) The effects of circLRBA on K48‐linked and K63‐linked polyubiquitination of Twist1 were analyzed in BC cells by Co‐IP and western blotting. G–I) The invasion and migration abilities of BC cells transfected or cotransfected with the indicated vectors and siRNAs were examined with Transwell assays with (G; invasion assays) or without Matrigel (H; migration assays), and wound healing (I) assays. (Magnification: Transwell assays, ×100; wound healing assay, ×50; scale bar: 200 µm for all). The data are presented as the mean ± SDs of at least three independent experiments. *p* < 0.05 was considered statistically significant. *p < 0.05, **p < 0.01, ***p < 0.001. Student's *t*‐test (A) and One‐way ANOVA (right panels of D, G, H, I) were used to determine statistical significance.

On the basis of these results, we hypothesized that circLRBA could promote EMT and metastasis by blocking the interaction between SPOP and Twist1. To prove this hypothesis, a cycloheximide (CHX) chase assay was used in BC cells to detect the effect of circLRBA on Twist1 stability. We found that overexpression of circLRBA significantly prolonged the half‐life of the Twist1 protein, whereas knockdown of circLRBA resulted in a sharp decrease in the protein level (**Figure** **7**A). Additionally, treatment with the proteasome inhibitor MG132 abolished the reduction in Twist1 protein levels mediated by circLRBA depletion (Figure 7B). Furthermore, the Co‐IP assay results indicated that fewer Twist1 proteins were immunoprecipitated by the anti‐SPOP antibody in MCF‐7 cells transfected with the circLRBA overexpression plasmid than in the control cells (Figure 7C), which suggested that the interaction between SPOP and Twist1 was interrupted by circLRBA, thereby inhibiting SPOP‐mediated Twist1 degradation. Moreover, western blot analysis showed that the overexpression or knockdown of SPOP could reverse the increase in or decrease in N‐cadherin, vimentin and Twist1 levels caused by the upregulation or downregulation of circLRBA, respectively, as well as rescue the E‐cadherin reduction or increase produced by circLRBA overexpression or knockdown (Figure 7D). The results of the ubiquitination assays indicated that upregulated or downregulated circLRBA suppressed or promoted Twist1 degradation in MDA‐MB‐231 cells, respectively, and the overexpression or depletion of SPOP reversed these effects (Figure 7E). Next, to clarify the lysine ubiquitination sites, upregulated circLRBA BC cells were treated with MG132, immunoprecipitated with anti‐Flag antibodies and immunoblotted with K48‐ and K63‐linked polyubiquitin antibodies. We found that circLRBA overexpression significantly suppressed both K48‐ and K63‐linked polyubiquitination of Twist1 (Figure 7F). Furthermore, the results of transwell and wound healing assays showed that the ectopic expression of SPOP could counteract the invasion‐ and migration‐ promoting effects induced by overexpression circLRBA, whereas the inhibitory effect of circLRBA depletion on BC cell invasion and migration abilities might be rescued by downregulating SPOP (Figure 7G–I). Together, these results suggest that circLRBA can competitively combine with SPOP to hinder the binding between SPOP and Twist1 and Twist1 ubiquitination, thereby promoting the invasion, migration and EMT of BC cells.

### 2.8 CircLRBA Knockdown Sensitizes BC Cells to DTX Treatment

**Figure 8:**
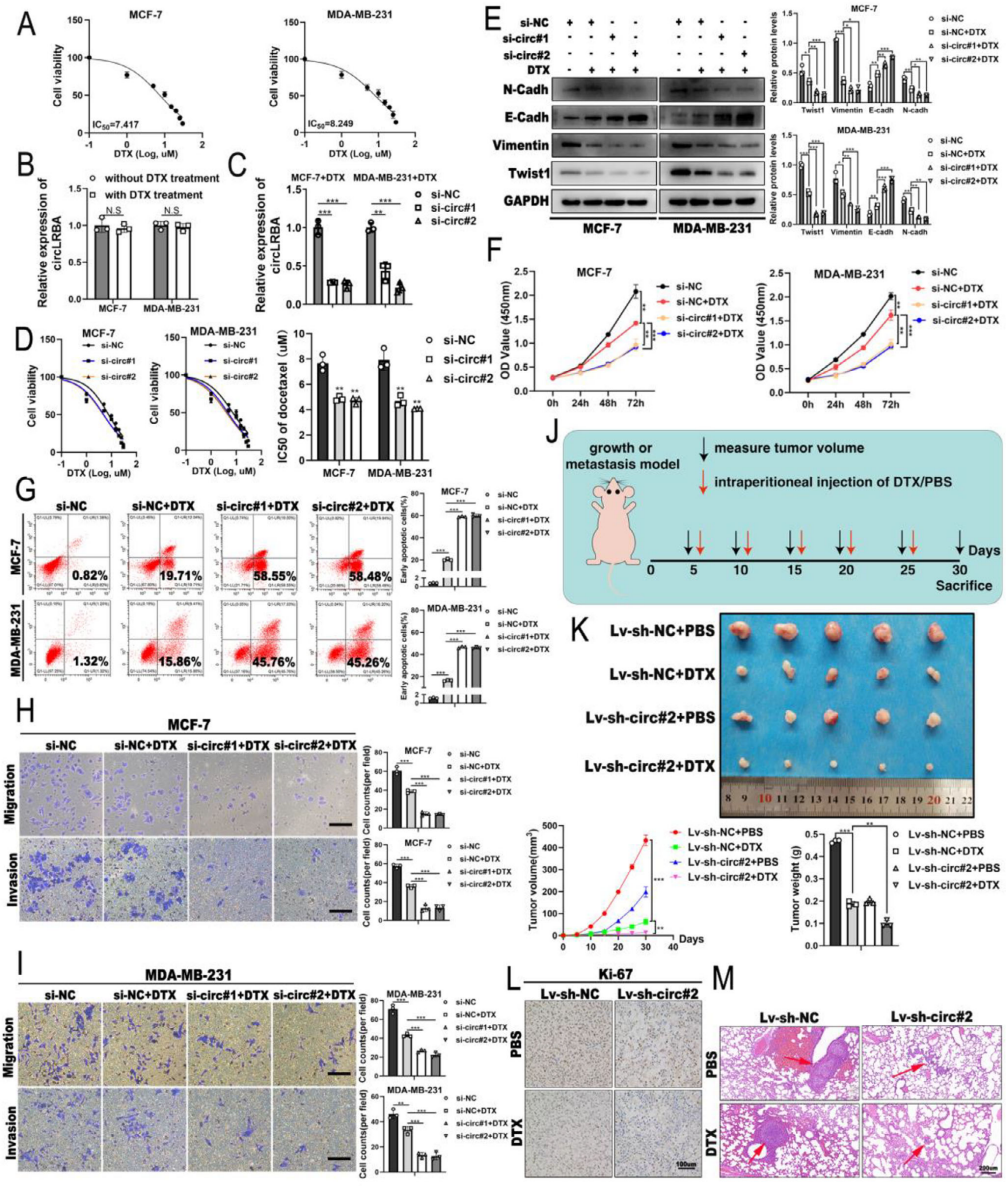
CircLRBA knockdown enhances the sensitivity of BC to DTX. A) The viability of BC cells treated with DTX was detected by the CCK‐8 assay. B) The expression of circLRBA in BC cells with or without DTX was measured by qRT‒PCR. C) The levels of circLRBA in BC cells transfected with siRNAs and treated with DTX were analyzed using qRT‒PCR. D) CCK‐8 assay results showing the viability of BC cells in each group treated with DTX. E) Western blot analysis of Twist1 and EMT‐related proteins in BC cells subjected to control conditions or circLRBA depletion, with or without DTX treatment. F–I) The growth (F), apoptosis (G), invasion and migration (H and I, magnification, ×100, scale bar, 200 µm) of BC cells treated with DTX were detected using functional assays. J) Illustrative diagram of the construction of the chemotherapy model. K) The volume and weight of the tumors in each group (n = 5 per group). L) IHC staining showing the expression levels of Ki‐67 in the indicated groups (magnification, ×200; scale bar, 100 µm). M) Representative images of lung metastatic nodules from each experimental group (n = 5 per group; magnification, ×100; scale bar, 200 µm). The data are presented as the mean ± SDs of at least three independent experiments. *p* < 0.05 was considered statistically significant. N.S., not significant, *p < 0.05, **p < 0.01, ***p < 0.001. Nonlinear Regression (A and D left panel), Student's *t*‐test (B) and One‐way ANOVA (C, D right panel, E right panel, F, right panels of G, H, I and K bottom panels) were used to determine statistical significance.

Previous studies have demonstrated that Twist1‐mediated EMT contributes to DTX resistance in BC.^[^ ^24^ ^]^ Our findings revealed that circLRBA enhances Twist1 stabilization and promotes EMT. To investigate whether circLRBA increases resistance to DTX in BC, BC cells were treated with different concentrations of DTX, and the IC_50_ was determined using the CCK‐8 assay (**Figure** **8**A). The effect of DTX on circLRBA expression was subsequently detected in BC cells, and qRT‒PCR analysis indicated that DTX did not alter the expression of circLRBA (Figure 8B), whereas circLRBA siRNAs significantly downregulated circLRBA levels in DTX‐treated BC cells (Figure 8C). The results of the CCK‐8 assay revealed that the resistance of BC cells to DTX was markedly decreased after circLRBA knockdown (Figure 8D). Furthermore, western blot analysis was performed to assess the expression levels of Twist1 and EMT‐related proteins in BC cells transfected with circLRBA siRNAs and treated with DTX. The data indicated that DTX treatment synergistically reduced the protein levels of Twist1, vimentin, and N‐cadherin while increasing E‐cadherin expression (Figure 8E). A series of functional assays were conducted to further investigate the role of circLRBA in DTX resistance. The results of the CCK‐8, flow cytometry analysis and Transwell assays demonstrated that DTX inhibited the proliferation, invasion, and migration of BC cells and induced apoptosis, while circLRBA silencing further synergistically enhanced these effects (Figure 8F–I). In vivo assays demonstrated that tumor growth in the circLRBA‐silenced group treated with DTX was significantly suppressed compared with that in the control groups (Figure 8J,K). IHC of the mouse tumor sections showed that compared with control treatment, circLRBA knockdown significantly reduced Ki‐67 expression in DTX‐treated tumors (Figure 8L). An experimental pulmonary metastasis assay was conducted. The results revealed that the mice injected with circLRBA‐silenced cells and treated with DTX presented fewer pulmonary metastatic nodules than the control mice (Figure 8M). Collectively, the above analyses demonstrated that circLRBA might promote the resistance of BC to DTX via Twist1‐induced EMT.

### 2.9 CircLRBA Suppresses CD8+ T Cell Infiltration and Promotes Immune Escape by Enhancing Twist1‐Mediated PD‐L1 Transcriptional Activity

**Figure 9:**
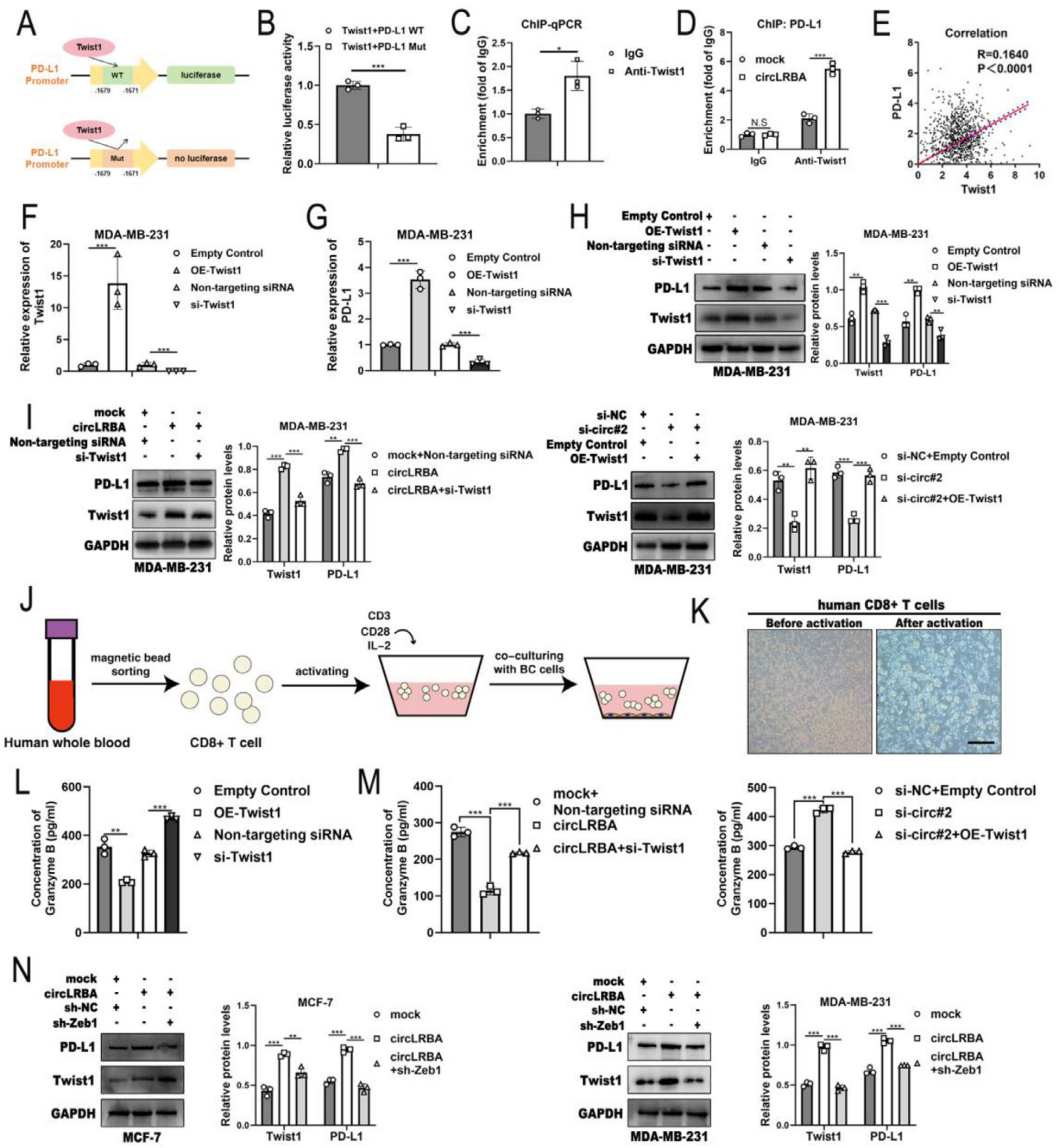
CircLRBA enhances Twist1‐mediated transcriptional activation of PD‐L1. A) Schematic model showing the hypothetical sites of Twist1 in the PD‐L1 gene promoter region. B) Twist1 binding to the PD‐L1 promoter region was analyzed using a dual‐luciferase reporter assay. C) ChIP‐qPCR confirmed the binding of Twist1 to the PD‐L1 promoter region. D) The binding of Twist1 to the PD‐L1 promoter after circLRBA upregulation was detected by ChIP followed by qPCR. E) The correlation between Twist1 and PD‐L1 expression in BC was analyzed using Pearson correlation analysis with data from the TCGA database. F) The efficiency of Twist1 overexpression plasmids and siRNAs targeting Twist1 was validated by qRT‒PCR (“Empty Control” indicates empty vector transfection; “Non‐targeting siRNA” denotes a negative control for siRNA‐mediated Twist1 silencing). G,H) The mRNA and protein levels of PD‐L1 in MDA‐MB‐231 cells following Twist1 overexpression or knockdown were analyzed by qRT‒PCR (G) and western blotting (H), respectively. I) The protein levels of Twist1 and PD‐L1 in BC cells transfected with the indicated plasmids and siRNAs were detected by western blotting. J) Illustrative diagram of the isolation and activation of human CD8+ T cells. K) Representative morphology images of CD8+ T cells before and after activation (magnification, ×100; scale bar, 200 µm). L,M) The concentration of Granzyme B secreted by CD8+ T cells into the supernatants was measured by ELISA. N) The protein levels of Twist1 and PD‐L1 in BC cells after circLRBA overexpression and Zeb1 knockdown were detected by western blotting. The data are presented as the mean ± SDs of at least three independent experiments. *p* < 0.05 was considered statistically significant. N.S., not significant, *p < 0.05, **p < 0.01, ***p < 0.001. Student's *t*‐test (B, C, D, F, G, H right panel and L), Pearson correlation analysis (E) and One‐way ANOVA (I, M and N) were used to determine statistical significance.

A previous study demonstrated that Twist1 could stimulate PD‐L1 transcription.^[^ ^25^ ^]^ Given the fact that circLRBA could increase Twist1 levels by stabilizing the Twist1 protein, we investigated whether circLRBA facilitates the transcriptional activation of PD‐L1 and immune evasion through regulating Twist1. We first predicted potential Twist1 binding sites on the PD‐L1 promoter using the JASPAR database and selected the site with the highest score for validation. Subsequent dual‐luciferase reporter assays and ChIP confirmed that Twist1 could directly bind to the PD‐L1 promoter (**Figure** **9**A–C). We further investigated the effect of circLRBA on PD‐L1 transcriptional activity. ChIP‐qPCR analysis revealed that the binding of Twist1 to the PD‐L1 promoter was enhanced after circLRBA overexpression (Figure 9D), suggesting that circLRBA might promote PD‐L1 expression at the transcriptional level via the regulation of Twist1. Moreover, analysis of the TCGA database revealed a significant positive correlation between Twist1 expression and PD‐L1 levels in BC (Figure 9E). We subsequently synthesized Twist1 overexpression plasmids and Twist1‐targeting siRNAs, and their efficacy was validated by qRT‒PCR (Figure 9F). The data showed that PD‐L1 mRNA and protein levels were significantly increased or decreased after Twist1 overexpression or knockdown, respectively (Figure 9G,H). Furthermore, circLRBA upregulation counteracted the decrease in the PD‐L1 level induced by Twist1 knockdown, whereas circLRBA depletion reversed the increase in the PD‐L1 level caused by Twist1 overexpression (Figure 9I). Next, CD8+ T cells from human whole blood were isolated and activated in vitro to explore the effect of circLRBA on the function of CD8+ T cells (Figure 9J). Activated CD8+ T cells presented increased cell volumes and cell clusters (Figure 9K). A coculture assay with MDA‐MB‐231 cells showed that the concentration of Granzyme B decreased or increased after Twist1 overexpression or knockdown, respectively, according to ELISA (Figure 9L). CircLRBA upregulation reversed the increase in Granzyme B levels induced by Twist1 knockdown, whereas circLRBA silencing reversed the decrease in Granzyme B levels caused by Twist1 overexpression (Figure 9M). We found that the transcription factor Zeb1 promoted circLRBA expression in breast cancer cells. To demonstrate that the phenotypic effects attributed to Zeb1 require circLRBA, we overexpressed circLRBA while silencing Zeb1 and measured the expression levels of Twist1 and PD‐L1. The results showed that Zeb1 knockdown reversed the upregulation of Twist1 and PD‐L1 caused by circLRBA overexpression, suggesting that circLRBA plays a critical role in this pathway (Figure 9N).

**Figure 10:**
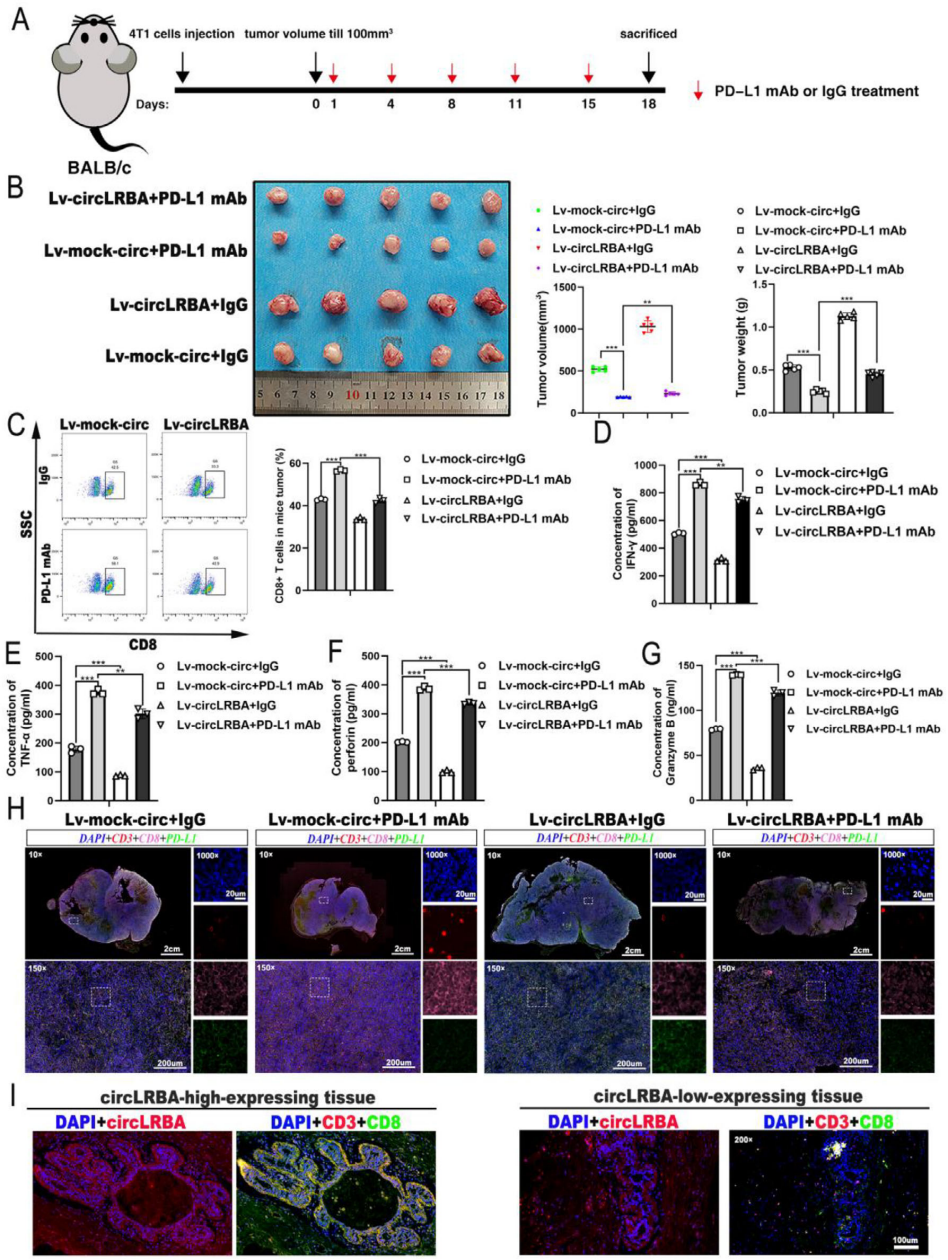
CircLRBA promotes resistance to anti‐PD‐L1 mAb therapy in vivo. A) Schematic diagram illustrating the establishment of the immunotherapy model. B) Tumor volume and weight in different treatment groups (n = 5 per group). C) The number of CD8+ T cells in tumors from mice treated with the indicated therapy was analyzed by flow cytometry. D–G) The levels of IFN‐γ (D), TNF‐α (E), Perforin (F) and Granzyme B (G) secreted by CD8+ T cells in mouse tumors were detected using ELISA. H) Representative mIHC images showing CD3, CD8, and PD‐L1 expression levels in the indicated treatment groups. I) The expression of circLRBA and the abundance of CD8+ T cells were assessed using combined FISH and IF staining (magnification, ×200; scale bar, 100 µm). The data are presented as the mean ± SDs of at least three independent experiments. *p* < 0.05 was considered statistically significant. *p < 0.05, **p < 0.01, ***p < 0.001. One‐way ANOVA (right panels of B and C, D, E, F, G) was used to determine statistical significance.

Moreover, to investigate the role of circLRBA in anti‐PD‐L1 therapy efficacy, we established a BALB/c mouse model via subcutaneous injection of the murine triple‐negative breast cancer cell line 4T1. Subsequently, the mice were treated with an anti‐PD‐L1 antibody or isotype control IgG via intraperitoneal injection at the indicated time points (**Figure** **10**A). Compared with the IgG control, anti‐PD‐L1 mAb therapy significantly suppressed tumor growth, and circLRBA overexpression in mice led to resistance to PD‐L1 mAb therapy (Figure 10B). The flow cytometry results demonstrated that the anti‐PD‐L1 antibody therapy group exhibited markedly greater CD8+ T cell numbers than did the control group, and circLRBA overexpression reduced CD8+ T cell infiltration into tumors (Figure 10C). Next, supernatants from mice tumor lysates were analyzed by ELISA, and the results indicated that the concentrations of IFN‐γ, TNF‐α, Perforin and Granzyme B were higher in the anti‐PD‐L1 treatment group than in the isotype control IgG group, while circLRBA overexpression suppressed the secretion of these factors (Figure 10D–G). mIHC revealed that PD‐L1 expression was reduced in the PD‐L1 mAb‐treated group, whereas circLRBA upregulation promoted PD‐L1 expression and inhibited CD8+ T cell infiltration (Figure 10H). Moreover, IF staining combined with FISH of human TNBC tissues revealed that tissues with high circLRBA expression contained significantly fewer CD8+ T cells than those with low circLRBA expression (Figure 10I). Taken together, these results suggest that circLRBA facilitates Twist1‐mediated transcriptional activation of PD‐L1 and suppresses the infiltration of CD8+ T cells, thus leading to immune evasion in BC.

## 3 Discussion

**Figure 11:**
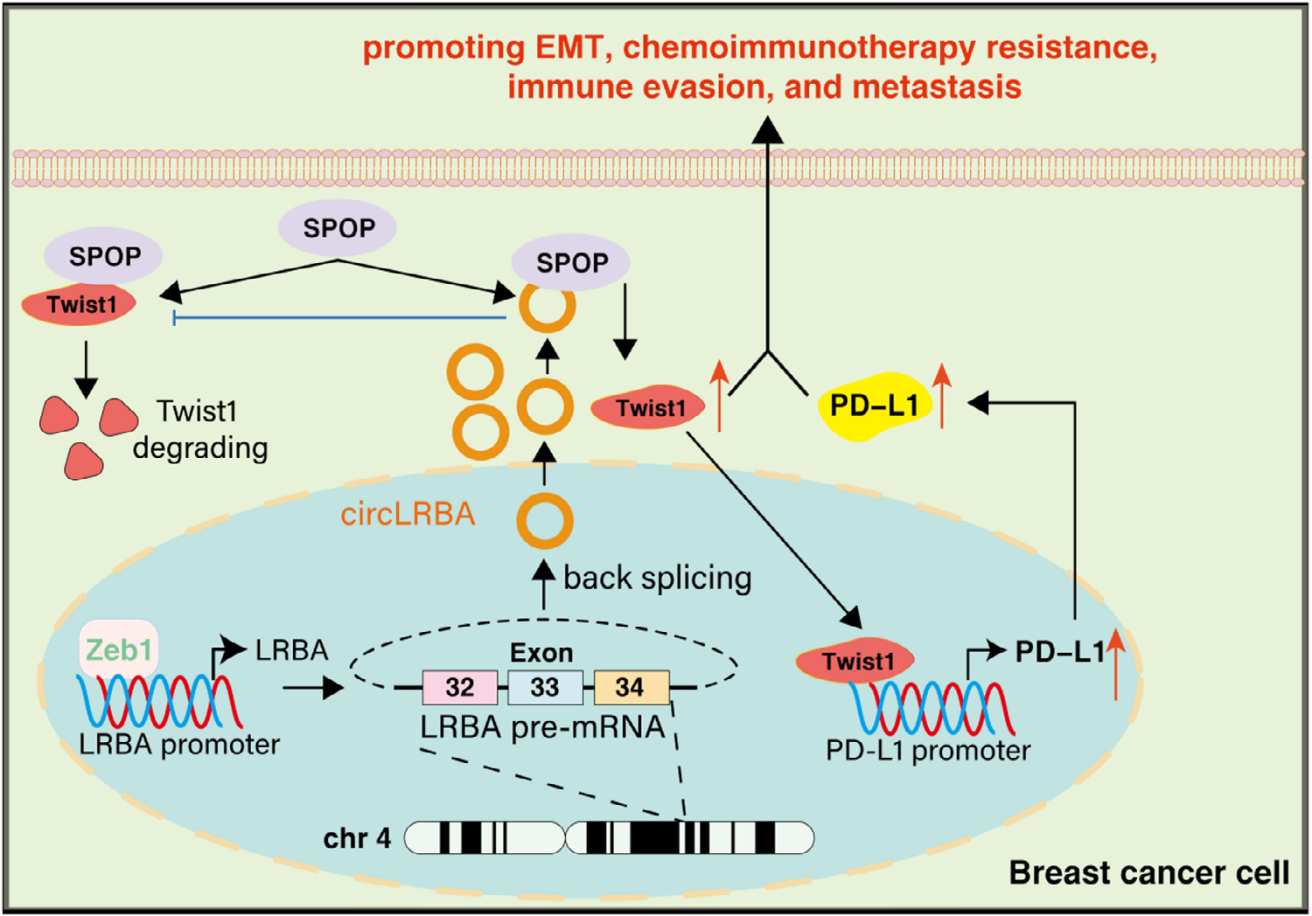
Schematic diagram illustrating the mechanism by which circLRBA promotes EMT, immune evasion, chemoimmunotherapy resistance and metastasis in BC.

Although significant progress has been achieved in the diagnosis and treatment of BC, the prognosis of patients with metastasis remains unsatisfactory. This poor outcome is partly attributable to the limited efficacy of conventional therapies such as chemotherapy and immunotherapy against distant metastases. An increasing number of studies have demonstrated that circRNAs play important roles in the development and metastasis of cancers.^[^ ^4^ ^]^ To further investigate the relationship between circRNAs and the progression of BC, in this study, we identified a previously uncharacterized circRNA circLRBA via RNA sequencing. We found that circLRBA was highly expressed in BC tissues and cells and closely correlated with lower overall survival and advanced pathological stages. Furthermore, circLRBA knockdown markedly inhibited the proliferation, migration, invasion, EMT, chemoimmunotherapy resistance, immune evasion and metastasis of BC cells both in vitro and in vivo. Mechanistically, the transcription factor Zeb1 promoted the generation of circLRBA. Furthermore, we demonstrated that circLRBA could interact competitively with the E3 ubiquitin ligase SPOP to hinder the binding between SPOP and Twist1 and enhance the transcriptional activation of PD‐L1, thus promoting the EMT, chemoimmunotherapy resistance and metastasis of breast cancer (**Figure** **11**).

The transcription factor Zeb1 is a member of the Zeb (zinc finger E‐box‐binding homeobox) family. Moreover, similar to Twist1, Zeb1 can also promote EMT by suppressing E‐cadherin protein levels.^[^ ^27^ ^]^ Previous studies have shown that Zeb1 plays vital roles in cancer progression. Jiang et al. reported that Zeb1 is highly expressed in breast cancer and increases growth, cancer metastasis, and chemoresistance by inducing aerobic glycolysis.^[^ ^28^ ^]^ Here, bioinformatic analysis and ChIP and luciferase reporter assays demonstrated that Zeb1 could bind to the LRBA gene promoter. The overexpression of Zeb1 subsequently enhanced the expression of circLRBA, while the knockdown of Zeb1 reduced the level of circLRBA, which suggested that Zeb1 might facilitate LRBA gene transcription and cooperate with circLRBA to promote EMT. Recent research has demonstrated that the transcription factor Zeb1 promotes the progression of epithelial ovarian cancer through enhancing the transcription of circANKRD17.^[^ ^29^ ^]^ These findings support our experimental results.

Several circRNAs are involved in EMT and metastasis in BC. For example, circEZH2 could interact with miR‐217‐5p to increase the expression of KLF5, thus upregulating CXCR4 and accelerating EMT in BC.^[^ ^30^ ^]^ Wang et al. reported that circNOL10 actives the JAK2/STAT5 pathway in BC by sponging miR‐767‐5p and thus suppressing EMT, migration and invasion.^[^ ^31^ ^]^ In addition, circEGFR promotes the EMT of triple‐negative breast cancer via the absorption of miR‐1299.^[^ ^32^ ^]^ Furthermore, circRNAs could also facilitate chemoimmunotherapy resistance and immune evasion in BC. Wang et al. demonstrated that circRNA‐CREIT promotes doxorubicin sensitivity in TNBC cells by binding to DHX9 and destabilizing PKR.^[^ ^33^ ^]^ CircBACH1 was shown to enhance the PTX chemoresistance of BC cells by sponging miR‐217 to upregulate the expression of G3BP2.^[^ ^34^ ^]^ Moreover, Liang et al. reported that circGSK3β facilitates PD‐L1 transcription and immune evasion in BC via the miR‐338‐3p/PRMT5/H3K4me3 axis.^[^ ^35^ ^]^ In our study, circLRBA could competitively bind to the E3 ubiquitin ligase SPOP to inhibit the interaction between SPOP and Twist1 and the degradation of Twist1. We also demonstrated that circLRBA suppresses the infiltration of CD8+ T cells by promoting Twist1‐mediated PD‐L1 transcription and expression. Our study may provide new insights into the regulation of chemoimmunotherapy resistance, immune evasion and metastasis, in BC through circRNA‐mediated regulation of EMT.

On the basis of the results of RNA pull down, mass spectrometry and RIP, we found that circLRBA could physically combine with SPOP in breast cancer cells. SPOP, a substrate binding adaptor of the E3 ubiquitin ligase Cullin3, is involved in cell growth, survival and signaling through the SPOP‐mediated ubiquitination of target substrates.^[^ ^36^ ^]^ It has been reported that SPOP promotes the ubiquitination and degradation of c‐myc and thus inhibits EMT by suppressing MTDH‐Twist1 signaling in BC.^[^ ^37^ ^]^ Conversely, another study revealed that the Cul3‐SPOP E3 ubiquitin ligase complex could promote metastasis through destabilizing breast cancer metastasis suppressor 1 (BRMS1) in BC.^[^ ^38^ ^]^ These studies showed that SPOP plays a dual role in BC progression through the targeting of diverse substrates. In the present study, we showed that SPOP was expressed at low levels in BC tissues. Moreover, the enforced expression of SPOP increased E‐cadherin expression and decreased N‐cadherin, vimentin and Twist1 protein levels in BC cells. We subsequently demonstrated that the N‐terminus of the MATH domain of SPOP could physically interact with Twist1 and promote its ubiquitination. Furthermore, SPOP overexpression significantly suppressed tumor metastasis in a mouse model. Previous studies have demonstrated that Twist1 plays an important role in the EMT and metastasis of tumor cells. Shibue et al. confirmed that Twist1 regulates the transcription of EMT‐related genes to promote cancer metastasis, such as downregulating the epithelial phenotype‐related gene E‐cadherin and upregulating the mesenchymal phenotype‐related gene vimentin.^[^ ^39^ ^]^ To date, only a few circRNAs have been implicated in EMT and metastasis through the regulation of Twist1.^[^ ^40^, ^41^ ^]^ Moreover, a previous study reported that SPOP inhibits EMT and metastasis by destabilizing Twist1 in breast cancer cells.^[^ ^26^ ^]^ Our results are consistent with these findings, and we demonstrated that circLRBA could increase Twist1 protein stability in BC cells through suppressing the binding of SPOP and Twist1 and Twist1 ubiquitination. Rescue experiments further confirmed that circLRBA promoted invasion, migration and EMT in BC cells through the suppression of Twist1 ubiquitination‐mediated degradation via interaction with SPOP.

In addition to promoting metastasis, ectopic Twist1 expression also induces chemoimmunotherapy resistance and immune evasion. Studies have revealed that Twist1 is involved in resistance to cisplatin in ovarian cancer,^[^ ^42^ ^]^ decitabine in acute myeloid leukemia ^[^ ^43^ ^]^ and pirarubicin in bladder cancer.^[^ ^44^ ^]^ Xu et al. demonstrated that Twist1 plays a critical role in DTX resistance by promoting EMT in BC.^[^ ^24^ ^]^ Here, we found that the proliferation, invasion and migration of BC cells were inhibited after DTX treatment and that circLRBA silencing synergistically enhanced these antitumor effects. In addition, Yang et al. demonstrated that Twist1 promotes the formation of an immunosuppressive tumor microenvironment via the SATB1/OPN axis and that Twist1 knockout could reverse tumor immune suppression and CAR T‐cell immunotherapy resistance.^[^ ^45^ ^]^ Twist1 promotes the transcription and expression of PD‐L1.^[^ ^25^ ^]^ PD‐L1 on the surface of cancer cells delivers inhibitory signals to T‐cells by binding to the PD‐1 receptor, thus inducing T cell exhaustion and dysfunction.^[^ ^46^ ^]^ Our results are consistent with these findings, and we further demonstrated that circLRBA could promote Twist1‐mediated transcriptional activation of PD‐L1, thus inhibiting the infiltration of CD8+ T cells and leading to immune evasion and chemoimmunotherapy resistance in BC.

Taken together, the results of our study reveal that circLRBA plays an oncogenic role in BC progression. We revealed that Zeb1 promotes circLRBA transcription. We further discovered that circLRBA can interact with SPOP to impair the binding between SPOP and Twist1, thus suppressing the SPOP‐mediated degradation of Twist1 and resulting in EMT, metastasis and chemoresistance in BC. Furthermore, circLRBA enhances PD‐L1 transcription via Twist1‐mediated activation, suppressing CD8+ T‐cell infiltration and facilitating immune evasion. Our work provides new insights into the mechanism of BC progression, suggesting that circLRBA might be a new prognostic indicator and potential therapeutic target for BC.

## 4 Experimental Section

### Patients and Samples

A total of 80 cancer tissue samples and matched paracancerous tissues were obtained from BC patients who underwent surgical therapy at the First Affiliated Hospital of Chongqing Medical University (Chongqing, China). All patients did not receive preoperative treatment and all tissues used in this study were collected with informed consent. The samples were stored in liquid nitrogen until use. The studies involving human tissue samples and the animal study protocol were approved by the Ethics Committee of the First Affiliated Hospital of Chongqing Medical University (Approval No. 2022–k228). The present study was conducted in accordance with the principles and guidelines of the Declaration of Helsinki.

### Cell Lines

All human BC cell lines, MCF‐7 (RRID: CVCL_0031), MDA‐MB‐231 (RRID: CVCL_0062), SK‐BR‐3 (RRID: CVCL_0033) and BT‐549 (RRID: CVCL_1092), the normal mammary epithelial cell line MCF‐10A (RRID: CVCL_0598), the human embryonic kidney 293T cell line (RRID: CVCL_0063) and the mouse BC cell line 4T1 (RRID: CVCL_0125), were purchased from the American Type Culture Collection (ATCC, USA). All the cell lines were confirmed to be mycoplasma free. MCF‐7 and MDA‐MB‐231 cells were maintained in DMEM (Gibco, New York, USA) supplemented with 10% fetal bovine serum (FBS; Gibco, New York, USA). BT‐549, SK‐BR‐3, 293T and 4T1 cells were cultivated in RPMI‐1640 medium (Gibco, New York, USA) containing 10% FBS. A MEBM Bullet Kit (Lonza, Basel, Switzerland) was used to culture the MCF‐10A cells. All the cell lines were cultured at 37 °C in a humid atmosphere with 5% CO_2_.

### Reverse Transcription PCR and Quantitative Real‐Time PCR

TRIzol reagent (Takara, Dalian, China) was used to extract total RNA, and reverse transcription (RT) was conducted using HiScript II Q RT SuperMix for qPCR (Vazyme, Nanjing, China) on a T100 Thermal Cycler instrument (Bio‐Rad, California, USA). The reverse transcription product was subsequently amplified with TB Green Premix Ex Taq (Takara, Dalian, China) in accordance with the manufacturer's instructions. The relative expression levels of circRNAs and mRNAs were calculated via the 2^–ΔΔCT^ method. All circLRBA detection experiments in this study were performed with validated divergent primers. GAPDH or U6 was used as an internal control. The primers used in these assays were described in Table S1, Supporting Information.

### RNA Sequencing

Total RNA was extracted from 4 pairs of cancer and matched normal tissues via TRIzol reagent. The quantity and quality of the RNAs were assessed by a NanoDrop ND‐1000 (Thermo Fisher Scientific, USA), and the linear RNAs were eliminated by RNase R. After RNA reverse transcription, the cDNA was amplified using the VAHTS Universal V6 RNA‐seq Library Prep Kit for Illumina (Vazyme, Nanjing, China). RNA sequencing was performed using the NovaSeq 6000 platform (*Illumina*, San Diego, CA, *USA*) from OE Biotechnology Co., Ltd. (Shanghai, China). From the RNA‐seq data, this work initially identified the most upregulated and downregulated circRNAs in breast cancer tissues compared with adjacent normal tissues, using the following criteria: |Fold change| ≥ 2 and *p <* 0.05.

### Plasmid Vector, Lentiviral Vector and siRNA Construction

Exons 32 to 34 of the LRBA gene were amplified and inserted into the plasmid pLC5‐ciR (Geneseed, Guangzhou, China) to construct a circLRBA overexpression vector, and a blank vector was used as a control. Two siRNAs targeting the backsplicing site of circLRBA and a negative control (si‐NC) were synthesized by Geneseed. Lentiviral vectors for overexpressing or knocking down circLRBA and SPOP were designed and synthesized by Genechem (Shanghai, China). The full‐length coding sequences of Zeb1, SPOP, and Twist1 were PCR‐amplified and cloned and inserted into the GV657 vector (CMV enhancer‐MCS‐3flag‐polyA‐EF1A‐zsGreen‐sv40‐puromycin), while shRNAs against Zeb1 and SPOP were cloned and inserted into the GV102 plasmid composed of hU6‐MCS‐CMV‐GFP ‐SV40‐Neomycin (*GeneChem*, *Shanghai, China*). siRNAs targeting Twist1 were chemically synthesized by Tsingke (Beijing, China). FLAG‐tagged Twist1, full‐length SPOP cDNA, and SPOP‐N/SPOP‐C domain fragments were subcloned and inserted into the pcDNA3.1 vector by GeneCreate (Wuhan, China). All the constructs were sequence‐verified. Neomycin or puromycin was used for screening stably transfected BC cells. All plasmids and siRNAs were transfected using Lipofectamine 3000 (Invitrogen, Carlsbad, CA, USA) according to the manufacturer's instructions. Briefly, cells were seeded in 6‐well plates at a density of 5 × 10^5^ cells well^−1^ and cultured to 70–80% confluence prior to transfection. The 2 µg plasmid or 50 nM siRNA was mixed with Lipofectamine 3000 diluted in DMEM (Gibco), and added to the cells, and then incubated at room temperature for 15 min. After 6 h, the transfection medium was replaced with complete culture medium. The sequences of all the shRNAs and siRNAs were showed in Table S2, Supporting Information.

### Actinomycin D and RNase R Treatment

BC cells were cultivated in DMEM supplemented with ACTD (3 ug mL^−1^; Sigma‐Aldrich, Steinheim, Germany), and the RNA was extracted with TRIzol reagent. For RNase R treatment, 500 ng of RNA from BC cells was digested with RNase R (1.0 U µg^−1^, Geneseed, Guangzhou, China), and then incubated for 20 min at 37 °C. After reverse transcription PCR, the expression levels of circLRBA and LRBA were determined using qRT‒PCR.

### Cytoplasmic/Nuclear RNA Fractionation

RNA from the cytoplasm and nucleus of BC cells was isolated according to the manufacturers’ protocols using a PARIS Kit (Invitrogen, USA), followed by reverse transcription and qRT‒PCR. U6 snRNA was used as a nuclear internal reference to standardize the expression of target RNA in nuclear fractions, while GAPDH mRNA was used as a cytoplasmic internal reference for normalization in cytoplasmic fractions. Relative expression levels were calculated via the 2^−ΔCt^ method.

### In Situ Hybridization

The probes used in the ISH were synthesized by Geneseed (Guangzhou, China), and the tissue microarray (TMA) chips were purchased from Outdo Biotech (Shanghai, China). For ISH, after being deparaffinized and dehydrated, the microarray chips were incubated with proteinase K at 37 °C for 20 min and treated with Triton‐X 100 at 4 °C for 10 min. Next, the chips were hybridized with a specific circLRBA probe, incubated with an anti‐digoxin antibody and stained with DAB. The expression scores of circLRBA were determined by multiplying the staining intensity (strong = 3, moderate = 2, weak = 1 and negative = 0) by the percentage of stained cells (<5% = 0, 5–25% = 1, 26–50% = 2, 51–75% = 3, >76% = 4). On the basis of the average score, the samples were classified into either a low‐expression group or a high‐expression group. The sequences of the ISH probes were listed in Table S3, Supporting Information.

### Fluorescence In Situ Hybridization and Immunofluorescence Co‐Staining

The BC cells on the coverslips were blocked with 2% BSA solution, and incubated with primary antibodies against SPOP (1:100 dilution, Proteintech, USA) and fluorescent secondary antibodies, and the cells were hybridized with digoxin‐tagged FISH probes for circLRBA (Geneseed, Guangzhou, China) and then stained with DAPI. Images were taken with a fluorescence microscope (Leica, Wetzlar, Germany). The sequence of the FISH probe was listed in Table S3, Supporting Information.

### Enzyme‐Linked ImmunoSorbent Assay

Supernatants from mice tumor lysates were diluted with the assay buffer provided in the ELISA kit (Mengbio, Chongqing, China). The concentrations of TNF‐α, IFN‐γ, Perforin and Granzyme B produced by CD8+ T cells within the tumor microenvironment were quantified according to the manufacturers' protocols. The optical density (OD) at 450 nm was measured using a microplate reader.

### DTX Treatment

BC cells were seeded into 96‐well plates at a density of 5 × 10^3^ cells per well and subsequently treated with different doses of DTX (0.1, 1, 5, 10, 15, 20, 25, and 30 µM) for 48 h. Cell viability and the half‐maximal inhibitory concentration (IC_50_) of DTX against BC cells were measured using the CCK‐8 assay.

For the mouse tumor model, mice with established tumors were randomly assigned to two treatment groups and received intraperitoneal injections of either PBS (vehicle) or DTX (10 mg kg^−1^) every 5 days for a total of six administrations.

### Isolation and Coculture of CD8 + T Cells

CD8+ T cells were isolated from healthy donor whole blood using the Human CD8 T Cell Isolation Kit (negative selection, BioLegend, San Diego, CA, USA) via a magnet (BioLegend, USA) according to the manufacturer's protocols. Isolated CD8+ T cells were cultured and activated in RPMI‐1640 medium supplemented with CD3 (2 µg mL^−1^, BioLegend, USA), CD28 (1 µg mL^−1^, BioLegend, USA) antibodies and IL‐2 (5 ng mL^−1^, BioLegend, USA) antibodies. Cancer cells were cocultured with activated CD8+ T cells at a 1:4 ratio (cancer cells: T cells) for 48 h under standard culture conditions, and then the supernatants were collected to detect granzyme B secretion via ELISA.

### Immunohistochemistry

For IHC, after dewaxing, rehydration and antigen retrieval, the tissue slides were incubated overnight with Twist1 (1:100 dilution, Proteintech, USA), E‐cadherin (1:200 dilution, CST, USA) and PD‐L1 (1:200 dilution, Proteintech, USA) primary antibodies at 4 °C. The samples were then incubated for 1 h with secondary antibodies and finally stained with diaminobenzidine (DAB) and hematoxylin. For multiplex immunohistochemistry (mIHC) of tissue sections, the slides were incubated with primary antibodies against CD3 (1:500 dilution, Servicebio, China), CD8 (1:200 dilution, Bioss, China), and PD‐L1 (1:100 dilution, Proteintech, USA) at 4 °C overnight. Subsequently, fluorochrome‐conjugated secondary antibodies were applied for 2 h at room temperature, followed by nuclear counterstaining with DAPI.

### Transwell and Wound Healing Assays

To distinguish migratory and invasive capacities, 2 × 10^4^ BC cells in 300 µL of serum‐free medium were seeded into the upper chambers of Transwell inserts (8 µm pores; Corning). For the migration assays, the inserts remained uncoated; for the invasion assays, the inserts were precoated with Matrigel (1:8 dilution in PBS, BD Biosciences, Bedford, MA, USA). The inserts were then transferred to 24‐well plates containing 700 µL of medium with 10% FBS in the bottom chambers, followed by a 24 h incubation at 37 °C with 5% CO_2_. For the wound healing assay, a 6‐well plate inoculated with BC cells was scratched with 200 µL tips, and the wound width was observed and recorded at 0 h and 24 h with a microscope (Leica, Germany).

### CCK‐8 and Colony Formation Assays

BC cell proliferation was quantified using the Cell Counting Kit‐8 assay (Beyotime, China). Briefly, the cells were seeded in 96‐well plates at 3000 cells well^−1^ in complete growth medium. Following 24 h of adherence, 10 µL of CCK‐8 reagent was added daily to triplicate wells, which were subsequently incubated for 2 h at 37 °C. The absorbance at 450 nm was measured using a microplate reader over four consecutive days. The cells were seeded for colony formation assays at 2000 cells well^−1^ in 6‐well plates. After 14 days in medium supplemented with 10% FBS, the colonies were methanol‐fixed, stained with crystal violet, and counted.

### Flow Cytometry Analysis

For detection of the cell cycle and apoptosis, PI staining and a Dead Cell Apoptosis Kit (Thermo Fisher Scientific, USA) were used before flow cytometry analysis.

To analyze CD8+ T cells in mouse tumor tissues, the samples were dissociated into single‐cell suspensions. The cells were subsequently stained with fluorochrome‐conjugated antibodies against CD3, CD8α, and CD45 (BioLegend, USA) and analyzed using a CytoFLEX flow cytometer (Beckman Coulter, CA, USA).

### Dual‐Luciferase Reporter Assay

The full‐length human Zeb1 gene was cloned and inserted into the pCDNA3.1 vector. Five pairs of wild‐type (Wt) and mutant type (Mut) putative sites of the LRBA promoter, along with one pair of Wt/Mut sites in the PD‐L1 promoter, were inserted into the pGL3‐Basic vector (Genecreate, Wuhan, China). These plasmids were cotransfected into 293T cells for 2 days. The firefly and Renilla luciferase activities were detected with a Dual Luciferase Report Assay System Kit (Promega, Madison, WI, USA) using GloMax 20/20 (Promega, USA).

### Ubiquitination Assay and CHX Chase Assay

For the ubiquitination assay, BC cells were transfected for 48 h and treated with 20 µM MG132 (MedChemExpress, Monmouth Junction, NJ, USA) for 12 h, after which the products were purified and collected via an immunoprecipitation (IP) assay with specific antibodies. For the CHX chase assay, after 48 h of transfection, BC cells were treated for 0, 2, 4, and 6 h with CHX (100 µg mL^−1^, Genview, Tallahassee, FL, USA). These samples were subsequently boiled at 100 °C for 10 min, after which western blotting was used to measure the protein levels.

### Animal Experiments

To investigate the role of circLRBA in tumor growth in vivo, 4‐week‐old female BALB/c nude mice from Tengxin Biotechnology Co., Ltd. (Chongqing, China) were subcutaneously injected with 1 × 10^7^ MDA‐MB‐231 cells transduced with a luciferase‐expressing lentivirus. These tumor‐bearing mice (n = 5 per group) were housed in individual ventilated cages for 4 weeks. Body weight and tumor volume were measured weekly. On the 28th day after inoculation with BC cells, the mice were euthanized. The tumors were subsequently removed, and the volume was calculated as length × width^2^ × 0.5. The slides from the tumors were prepared (Aochuang Biotechnology Co., Ltd., Chengdu, China) for further study. For survival studies, the mice (n = 10 per group) received tail vein injections of MDA‐MB‐231 cells (4 × 10^6^ cells per mouse) and were monitored for 60 days, and survival was recorded daily. For the metastasis assays, separate cohorts (n = 5 per group) received identical cell injections. On post‐injection day 28, the mice were injected intraperitoneally with D‐luciferin (150 mg kg^−1^) and imaged (NightOWL LB 983; Berthold). After euthanasia, the lungs were harvested, fixed in 4% paraformaldehyde (PFA), and H&E‐stained for metastatic nodule quantification.

### Anti‐PD‐L1 mAb Therapy

For the immunotherapy model, tumor‐bearing BALB/c mice were intraperitoneally administered either a mouse anti‐PD‐L1 mAb (100 µg, Bio X Cell, USA) or an isotype control IgG (100 µg, Bio X Cell, USA) twice weekly for a total of 5 injections. At 18 days post‐treatment initiation, the mice were euthanized, and the tumors were harvested and processed for subsequent analysis.

### Western Blotting

RIPA lysis buffer containing PMSF (Beyotime, Beijing, China) was used to extract BC cell proteins after transfection. Proteins were quantified using a BCA protein assay kit (Biosharp, Hefei, China), separated using SDS‐PAGE, and then transferred onto PVDF membranes (Millipore, Billerica, MA, USA). These membranes were blocked in 5% nonfat milk for 2 h and then incubated with primary antibodies against Bcl‐2 (1:1000 dilution, CST, USA), Bax (1:1000 dilution, Abcam, USA), Twist1 (1:1000 dilution, Proteintech, USA), SPOP (1:1000 dilution, Proteintech, USA), vimentin (1:5000 dilution, Proteintech, USA), E‐cadherin (1:1000 dilution, Proteintech, USA), N‐cadherin (1:1000 dilution, Proteintech, USA), K48‐linked polyubiquitin (1:1000 dilution, CST, USA), K63‐linked polyubiquitin (1:1000 dilution, CST, USA), PD‐L1 (1:1000 dilution, Proteintech, USA) and GAPDH (1:20 000 dilution, Proteintech, USA) as internal references overnight at 4 °C. Next, the membranes were incubated with HRP‐conjugated secondary antibodies (1:5000 dilution, Proteintech, USA) for 2 h at room temperature and visualized via Pierce ECL (Thermo Fisher Scientific, USA) using a ChemiDoc Imaging System (Bio‐Rad, California, USA).

### Pull Down and Mass Spectrometry

To validate the binding of circLRBA to proteins, a pair of biotin‐labeled probes, including a negative control and specific to the junction sites of circLRBA, were designed and synthesized by GeneCreate (Wuhan, China). The BC cell lysates were mixed with the probes for 16 h at room temperature and incubated with the streptavidin magnetic beads (Thermo Fisher Scientific, USA) at 4 °C overnight. The precipitated proteins were separated using SDS‐PAGE, and the gel was stained via a silver stain kit (Beyotime, Beijing, China). The proteins were retrieved and identified via mass spectrometry and western blotting. The probes used for pull‐down were listed in Table S3, Supporting Information.

### RNA Immunoprecipitation

The RIP assays for SPOP and circLRBA were carried out with an RNA immunoprecipitation kit (Geneseed, Guangzhou, China). In brief, the lysates of 1 × 10^7^ MCF‐7 cells were mixed with magnetic beads and a mixture of SPOP‐specific antibody (5 µg, Proteintech, USA) overnight at 4 °C. IgG antibody (5 µg, Proteintech, USA) was used as a negative control. The levels of precipitated RNA and SPOP proteins were determined via RT‒PCR, qRT‒PCR and western blotting, respectively.

### Co‐Immunoprecipitation

Co‐immunoprecipitation was performed using a Pierce Classic Magnetic IP/Co‐IP Kit (Thermo Fisher Scientific, USA). Briefly, 1 × 10^7^ MCF‐7 cells were lysed for 20 min on ice by IP lysis/wash buffer. After centrifugation, some lysates were frozen at −80 °C as an input positive control, and the other lysates were equally divided, incubated with 5 µg of SPOP and Twist1 antibodies overnight at 4 °C, and then incubated with Protein A/G Magnetic Beads for 4 h at 4 °C. The beads were collected via a magnetic stand, and the target proteins were eluted using IP lysis/wash buffer, and then subjected to western blotting.

### Chromatin Immunoprecipitation

The ChIP assay was conducted using a SimpleChIP Enzymatic Chromatin IP Kit (CST, USA) following the manufacturer's protocols. Specific antibodies against Zeb1 (Abcam, USA), Twist1 (Proteintech, USA) and IgG (Proteintech, USA) were used, and the DNA was eluted, purified and detected via qPCR. The primers used in this assay were described in Table S1, Supporting Information.

### Electrophoretic Mobility Shift Assay

For EMSA, specific oligonucleotide probes targeting circLRBA synthesized by Genecreate (Wuhan, China) were incubated for 30 min with nuclear protein extracts from BC cells at room temperature to allow protein‐RNA complex formation. To specifically confirm the binding of the SPOP protein to circLRBA, supershift assays were performed. Following the initial 30‐min binding reaction, anti‐SPOP antibody (1 µg, Proteintech, USA) was added to the reaction mixtures. The mixtures were incubated for 1 h at room temperature, and subjected to electrophoresis on a non‐denaturing 0.5 × TBE 6% polyacrylamide gel, followed by transfer onto a nylon membrane. Finally, the signal was visualized with a ChemiDoc XRS system(Bio‐Rad, California, USA). The probe sequences of the EMSA were listed in Table S3, Supporting Information.

### Statistical Analysis

Statistical analyses were performed with GraphPad Prism 10.0 (San Diego, CA, USA) and SPSS 22.0 (IBM, SPSS, Chicago, IL, USA). Differences between two groups or among multiple groups were analyzed by Student's *t*‐test or one‐way ANOVA, respectively. Relevance between groups was calculated using the χ^2^ test. The Kaplan–Meier method was applied to calculate survival curves. Cox proportional hazards regression models with univariate and multivariate analyses were applied to estimate independent prognostic factors. The diagnostic value of circLRBA was assessed via receiver operating characteristic (ROC) curves. Drug concentration data underwent log_10_ transformation followed by nonlinear regression analysis to generate dose‐response curves. Minimum triplicate independent experiments were performed. In vivo models (subcutaneous xenografts and experimental metastasis) utilized mice as biological replicates (n ≥ 5 per group). In vitro assays included triplicate technical measurements per biological sample across three biological replicates. The data were presented as the means ± standard deviations. The results were regarded as statistically significant when *p* < 0.05.

## Conflict of Interest

The authors declare no competing interests.

## Supporting information



Supporting Information

## Data Availability

The data that support the findings of this study are available from the corresponding author upon reasonable request.
