# Supplementary material for: CircLRBA Promotes Epithelial‐Mesenchymal Transition, Immune Evasion, Chemoimmunotherapy Resistance and Metastasis Through Stabilizing Twist1
Source: Adv Sci (Weinh). 2025 Oct 13;12(48):e08918. doi: 10.1002/advs.202508918 (PMC12752550; doi:10.1002/advs.202508918)
Supplement: Supplementary file 1 — Supporting Information [file ADVS-12-e08918-s001.docx]

**Supplementary Information for**

**CircLRBA Promotes Epithelial-Mesenchymal Transition, Immune Evasion, Chemoimmunotherapy Resistance and Metastasis through Stabilizing Twist1**

Xiaosong Wang^1^, Xin Yang^1^, Lei Xing^2^, Hang Chen^1,3^, Fuming Xie^1,3^, [Bin Wang](https://pubmed.ncbi.nlm.nih.gov/?term=Wang+B&cauthor_id=36895010)^4^, Bowen Shi^1^, Yan Yang^1^, Junxia Chen^1^*

^1^Department of Cell Biology and Genetics, Chongqing Medical University, Chongqing 400016, China.

^2^Department of Endocrine and breast surgery, The First Affiliated Hospital of Chongqing Medical University, Chongqing, 400016, China.

^3^Institute of Hepatopancreatobiliary Surgery, Chongqing General Hospital, Chongqing University, Chongqing, 401147, China.

^4^Department of Oncology, The Seventh People's Hospital of Chongqing, Chongqing， 400054, China.

*Correspondence:

Junxia Chen

E-mail: [chjunxia@126.com](mailto:chjunxia@126.com)

**Table S1. Sequences of primers used in this study.**

| **Gene** | **Primer sequences** |
| --- | --- |
| **CircLRBA** | F: 5’-AGCCATGGACAAGAACTGCT-3’ |
|  | R: 5’-ACTGTTGGAACTGAGGGCAA-3’ |
| **GAPDH** | F: 5’-GAAGGTGAAGGTCGGAGTC-3’ |
|  | R: 5’-GAAGATGGTGATGGGATTTC-3’ |
| **CircLRBA**  **(Convergent)** | F: 5’-GCTTTGGAAAAGGCAGCTCC-3’ |
|  | R: 5’-GGCTACCCAAAAGTGTCCGA-3’ |
| **LRBA** | F: 5’-CACAGACAAAGCCCAGCAGGATG-3’ |
|  | R: 5’-ATCACAGCGGGAGTTGCCAAAC-3’ |
| **U6** | F: 5'- GCTTCGGCAGCACATATACTAAAAT-3' |
|  | R: 5'- CGCTTCACGAATTTGCGTGTCAT-3' |
| **SPOP** | F: 5'-CATGGTAAAGGTTCCTGAGTGC-3' |
|  | R: 5'- AACACACAAGCAGCAGTCTG-3' |
| **Zeb1** | F: 5'- AACCCATAGTGGTTGCTTCAGG-3' |
|  | R: 5'- AACAACAGCTTGCACCATGC-3' |
| **LRBA (Promoter 2 site)** | F: 5'-GAGCACGGGTCTTGCTGTT-3' |
|  | F: 5'-AGGGAGGGCTGGGTGTAGT-3' |
| **Twist1** | F: 5’-GTACATCGACTTCCTCTACCAG-3’ |
|  | R: 5’-CATCCTCCAGACCGAGAAG-3’ |
| **PD-L1** | F: 5’-GTGCCGACTACAAGCGAATTAC-3’ |
|  | R: 5’-GGAATTGGTGGTGGTGGTCTTAC-3’ |
| **PD-L1**  **(for ChIP-qPCR)** | F: 5’-ACCTTAAGCTCTTACCCCTCTG-3’ |
|  | R: 5’-TTTCACCGGGAAGAGTTTCG-3’ |

**Table S2. Sequences of siRNAs and shRNAs used in this study.**

| **Definition** | **Sequences** |
| --- | --- |
| **si-NC** | 5’-TTCTCCGAACGTGTCACGT-3’ |
| **si-circ#1** | 5’-TGTGTTCTCAGGCAGTAGA-3’ |
| **si-circ#2** | 5’-GTGTTCTCAGGCAGTAGAT-3’ |
| **sh-Zeb1** | 5’-GATCCCGCTGTTGTTCTGCCAACAGTTCTCGAGAACTGTTGGCAGAACAACAGCTTTTTGGAT-3’ |
|  | 5’-AGCTATCCAAAAAGCTGTTGTTCTGCCAACAGTTCTCGAGAACTGTTGGCAGAACAACAGCGG-3’ |
| **sh-SPOP** | 5’-GATCCCGAGGTGAGTGTTGTGCAAGATCTCGAGATCTTGCACAACACTCACCTCTTTTTGGAT-3’ |
|  | 5’-AGCTATCCAAAAAGAGGTGAGTGTTGTGCAAGATCTCGAGATCTTGCACAACACTCACCTCGG-3’ |
| **NC-Twist1** | 5’-TTCTCCGAACGTGTCACGT-3’ |
|  | 5’-ACGTGACACGTTCGGAGAA-3’ |
| **si-Twist1** | 5’-AGTCTTACGAGGAGCTGCA-3’ |
|  | 5’-TGCAGCTCCTCGTAAGACT-3’ |

**Table S3. Sequences of probes used in this study.**

| **Method** | **Sequences** | |
| --- | --- | --- |
| **FISH** | 5’DIG-GATGATCTACTGCCTGAGAACAC- 3’-DIG | |
| **ISH** | 5’digoxin-GATGATCTACTGCCTGAGAACAC- 3’-digoxin | |
| **Pull-down** | **Control** | Biotin-TATCACGTAGCCGTTGCATTTGCCGTAGCCCTGTGGGCC |
|  | **CircLRBA** | Biotin-GCATTAGATGATCTACTGCCTGAGAACACAGTAGCATAA |
| **EMSA** | **ProbeA** | CCTCAGTTCCAACAGTTGATTCAGTTTCACAAGATCCGGTTTCAAATATGAGT |
|  | **ProbeB** | ATATGAGTATTACAGAGAGGCTTGAACACGCTTTGGAAAAGGCAGCTCCTCTC |
|  | **ProbeC** | CTCCTCTCCTTCGTGAGATTTTTGTGGATTTTGCACCTTTTCTTTCTCGGACA |
|  | **ProbeD** | CTCGGACACTTTTGGGTAGCCATGGACAAGAACTGCTTATAGAAGGAACAAGT |
|  | **ProbeE** | GAACAAGTCTGGTTTGCATGAAGTCGAGTAGTTCAGTTGTGGAATTGGTTATG |
|  | **ProbeF** | TGGTTATGCTACTGTGTTCTCAGGCAGTAGATCATCTAATGCAAAATTGCCCTCAGTT |


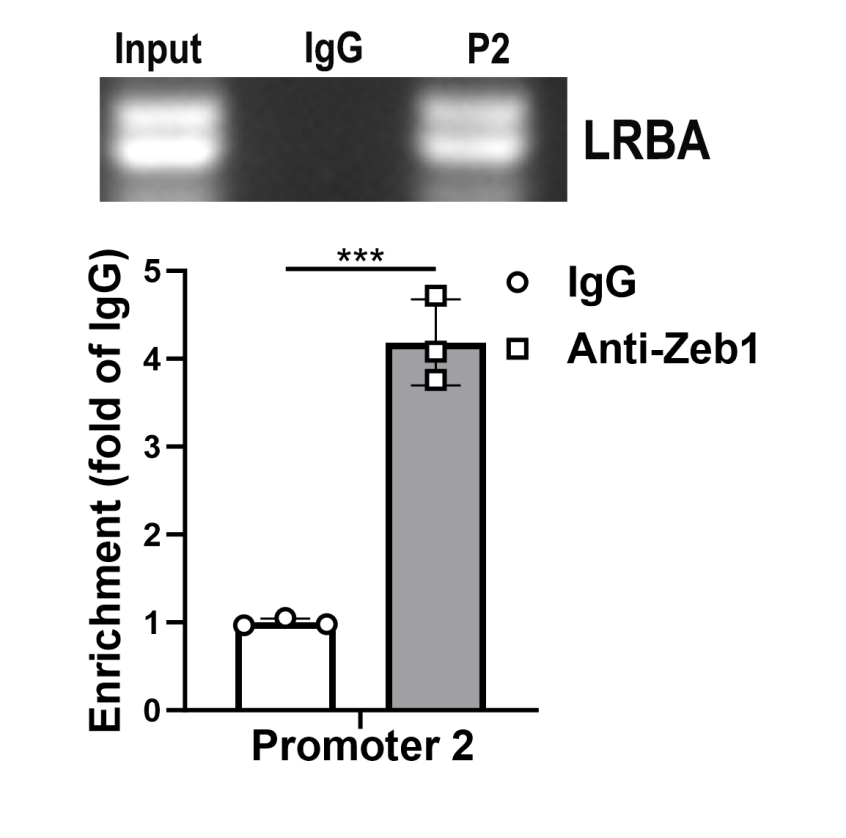


**Figure S1.** The binding between Zeb1 and LRBA promoter region in MDA-MB-231 cells was verified by ChIP-qPCR followed by RT-PCR. The data are presented as the mean ± SDs of at least three independent experiments. P < 0.05 was considered statistically significant. ***P < 0.001. Student’s t-test was used to determine statistical significance.


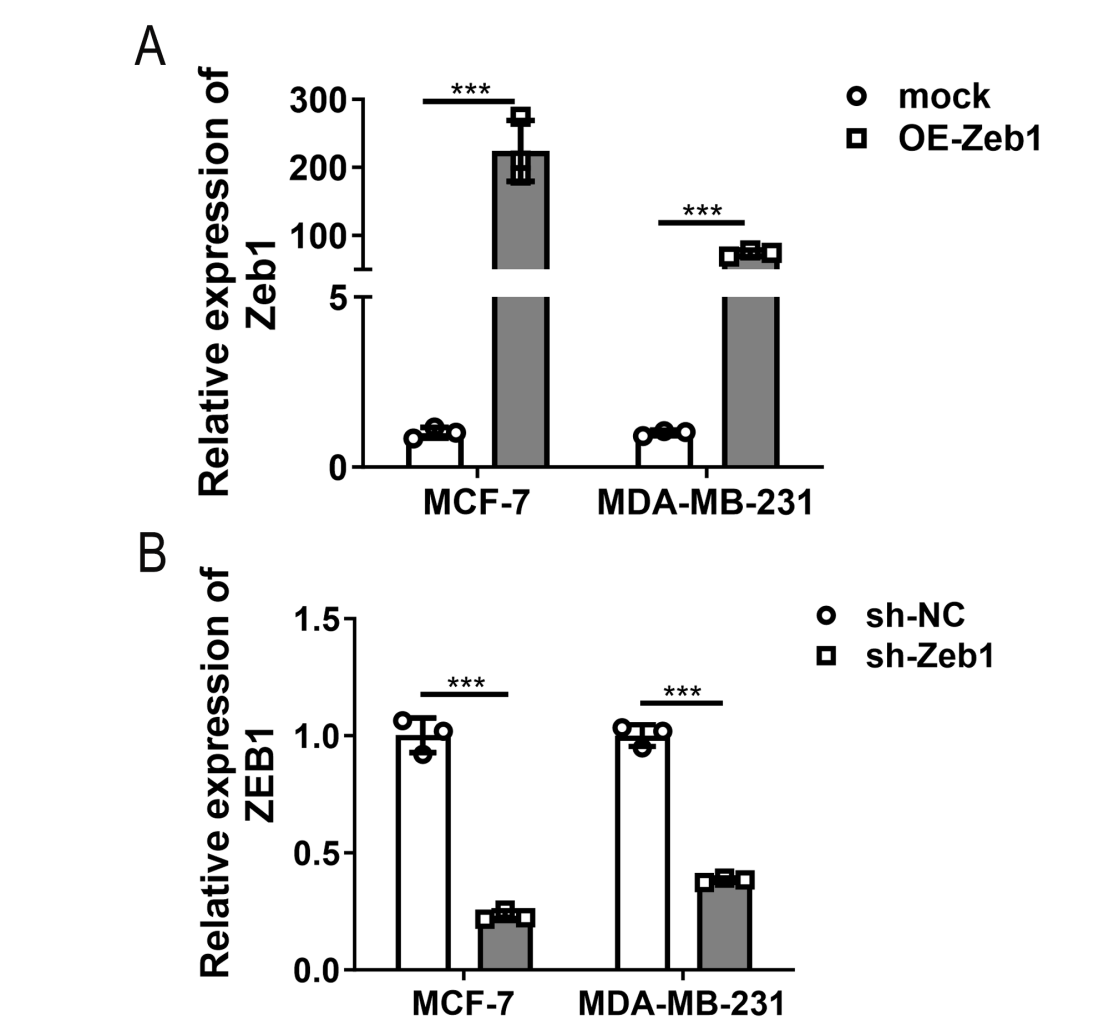


**Figure S2. Validation of Zeb1 overexpression and knockdown plasmids.** A-B) The the efficiency of the overexpression vector or siRNAs of Zeb1 in BC cells was detected. The data are presented as the mean ± SDs of at least three independent experiments. P < 0.05 was considered statistically significant. ****P < 0.001*. Student’s t-test (A and B) were used to determine statistical significance.


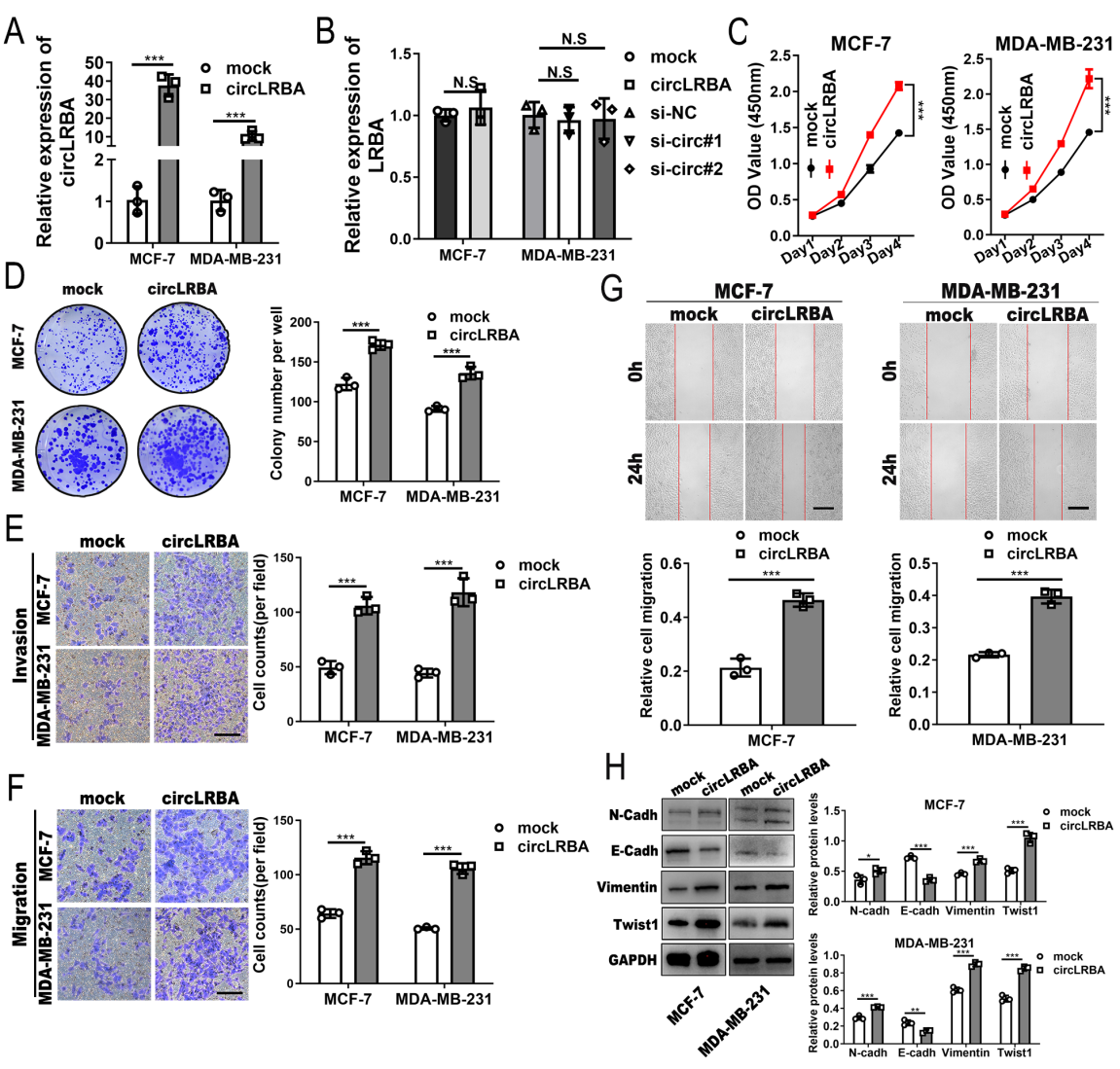


**Figure S3. CircLRBA knockdown inhibits the proliferation, invasion, migratio and EMT in BC.** A-B) The expressions of circLRBA and LRBA in BC cells were determined after overexpression and knockdown of circLRBA by qRT‒PCR. C-G) Overexpression of circLRBA increased proliferation, invasion and migration of BC cells by CCK-8 (C), colony formation (D), transwell (E, F) and wound healing (G) assays. H) The expression levels of EMT-related proteins in BC cells were detected after upregulation of circLRBA using western blot. The data are presented as the mean ± SDs of at least three independent experiments. P < 0.05 was considered statistically significant. **P < 0.05, **P < 0.01, ***P < 0.001*. Student’s t-test (A and C-G) and One-way ANOVA (B) were used to determine statistical significance.


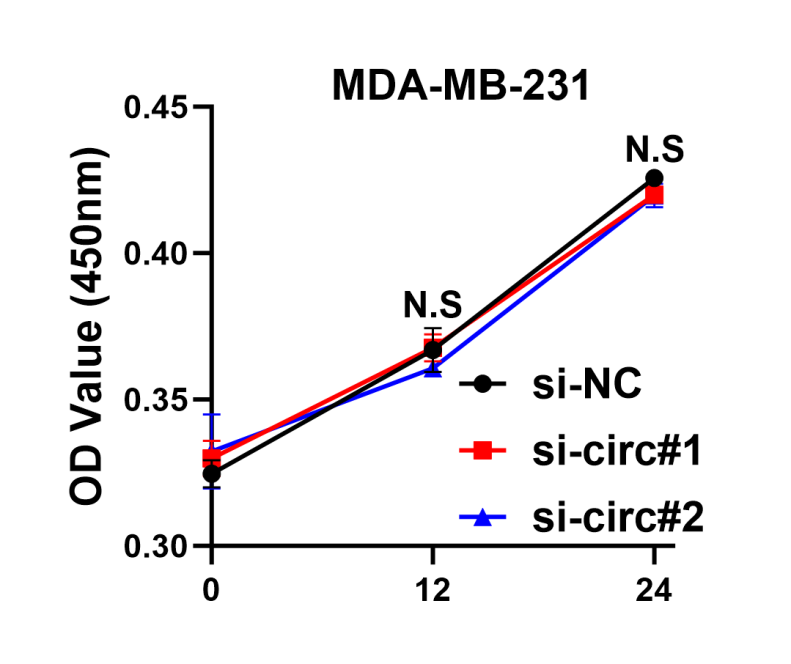


**Figure S4.** Cell proliferation of MDA-MB-231 cells was assessed using CCK-8 in parallel experiments. The data are presented as the mean ± SDs of at least three independent experiments. P < 0.05 was considered statistically significant. N.S., not significant. One-way ANOVA was used to determine statistical significance.


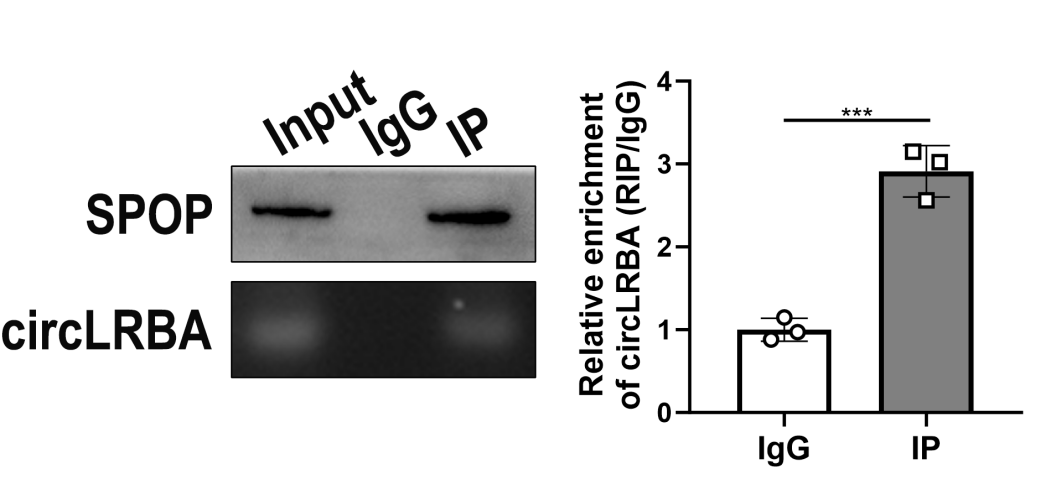


**Figure S5.** The interaction between circLRBA and SPOP in MDA-MB-231 cells was verified by RNA immunoprecipitation (RIP) followed by RT-PCR for circLRBA and western blot for SPOP. The data are presented as the mean ± SDs of at least three independent experiments. P < 0.05 was considered statistically significant. ***P < 0.001. Student’s t-test was used to determine statistical significance.


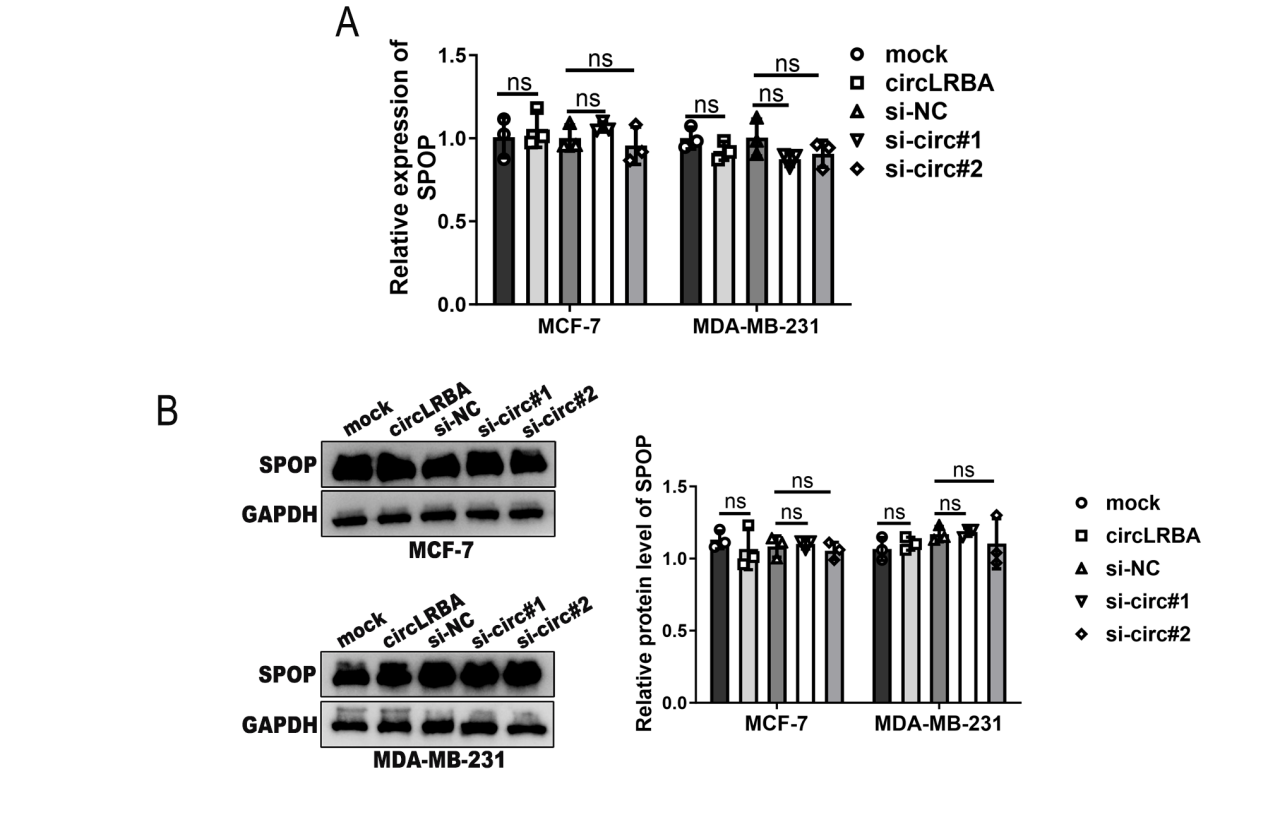


**Figure S6. The effect of circLRBA on the expression level of SPOP.** A-B) The expression level of SPOP was determined in BC cells after overexpression and knockdown of circLRBA by qRT‒PCR or western blot. The data are presented as the mean ± SDs of at least three independent experiments. P < 0.05 was considered statistically significant. ns, not significant. One-way ANOVA was used to determine statistical significance.
